# Construction of an interferon regulatory factors-related risk model for predicting prognosis, immune microenvironment and immunotherapy in clear cell renal cell carcinoma

**DOI:** 10.3389/fonc.2023.1131191

**Published:** 2023-04-27

**Authors:** Hao Pan, Wei Lu, Mengyuan Zhang, Chengxiao Liu

**Affiliations:** ^1^ Department of Anesthesiology, Shandong Provincial Hospital Affiliated to Shandong First Medical University, Jinan, China; ^2^ Department of Oral and Maxillofacial Surgery, Shandong Provincial Hospital Affiliated to Shandong First Medical University, Jinan, China

**Keywords:** interferon regulatory factors, clear cell renal cell carcinoma, tumor microenvironment, immunotherapy, drug sensitivity

## Abstract

**Background:**

Interferon regulatory factors (IRFs) played complex and essential roles in progression, prognosis, and immune microenvironment in clear cell renal cell carcinoma (ccRCC). The purpose of this study was to construct a novel IRFs-related risk model to predict prognosis, tumor microenvironment (TME) and immunotherapy response in ccRCC.

**Methods:**

Multi-omics analysis of IRFs in ccRCC was performed based on bulk RNA sequencing and single cell RNA sequencing data. According to the expression profiles of IRFs, the ccRCC samples were clustered by non-negative matrix factorization (NMF) algorithm. Then, least absolute shrinkage and selection operator (LASSO) and Cox regression analyses were applied to construct a risk model to predict prognosis, immune cells infiltration, immunotherapy response and targeted drug sensitivity in ccRCC. Furthermore, a nomogram comprising the risk model and clinical characteristics was established.

**Results:**

Two molecular subtypes with different prognosis, clinical characteristics and infiltration levels of immune cells were identified in ccRCC. The IRFs-related risk model was developed as an independent prognostic indicator in the TCGA-KIRC cohort and validated in the E-MTAB-1980 cohort. The overall survival of patients in the low-risk group was better than that in the high-risk group. The risk model was superior to clinical characteristics and the ClearCode34 model in predicting the prognosis. In addition, a nomogram was developed to improve the clinical utility of the risk model. Moreover, the high-risk group had higher infiltration levels of CD8^+^ T cell, macrophages, T follicular helper cells and T helper (Th1) cells and activity score of type I IFN response but lower infiltration levels of mast cells and activity score of type II IFN response. Cancer immunity cycle showed that the immune activity score of most steps was remarkably higher in the high-risk group. TIDE scores indicated that patients in the low-risk group were more likely responsive to immunotherapy. Patients in different risk groups showed diverse drug sensitivity to axitinib, sorafenib, gefitinib, erlotinib, dasatinib and rapamycin.

**Conclusions:**

In brief, a robust and effective risk model was developed to predict prognosis, TME characteristics and responses to immunotherapy and targeted drugs in ccRCC, which might provide new insights into personalized and precise therapeutic strategies.

## Introduction

Clear cell renal cell carcinoma (ccRCC) is the most common histological subtype of renal cell carcinoma and accounts for approximately 80%-90% of cases (1). Radical nephrectomy remains the effective option for localized ccRCC, however, nearly 30% of patients develop distant metastatic or recurrence after surgery (2, 3). TKIs-targeted and mTOR-targeted therapies have been widely adopted, but the clinical benefits are limited (4). In recent years, immune checkpoint inhibitors (ICIs) therapy targeting PD-1/PD-L1 and/or CTLA-4 has made significant breakthroughs in ccRCC (5, 6). However, the therapeutic response rate of ICIs in ccRCC remains poor (7). Despite the combination treatment of ICIs and targeted therapeutic drugs may improve the response rate, these patients receiving the combination therapy often suffer from adverse events (5, 8, 9). Moreover, ccRCC exhibits extremely high heterogeneity, so the responses and prognoses after immunotherapy in patients with the same degree of progression vary extensively (10). Therefore, it is essential to explore the heterogeneity of the ccRCC patients and develop novel biomarkers or therapeutic targets to predict the prognosis and improve ICIs therapeutic efficacy, thereby optimizing immunotherapy for ccRCC.

Interferon regulatory factors (IRFs), which comprise nine gene family members (IRF1, IRF2, IRF3, IRF4, IRF5, IRF6, IRF7, IRF8 and IRF9), are a family of transcription factors that regulate the transcription process of interferons by acting at their gene sites (11). Cumulative evidences revealed IRFs played critical roles in the regulation of cell cycle, cell differentiation, cell apoptosis and cancer immune responses (11). Multiple studies suggested that IRFs played complex and essential roles in progression, prognosis, and immune microenvironment in ccRCC. Kong et al. reported that PD-L1 expression in ccRCC cells was induced by IFNγ stimulation through activation of JAK2/STAT1/IRF1 signaling (12). In addition, the high expression of IRF3 and IRF4 was found to be significantly associated with the advanced clinical stage and poor prognosis in ccRCC (13, 14). Moreover, Bai et al. found high expression of IRF5 was significantly associated with poor overall survival (OS) and recurrence free survival (RFS) in ccRCC (15). Furthermore, Ma et al. revealed that IRF6 overexpression could attenuate proliferation, migration and invasion of ccRCC cells by downregulating the KIF20A expression (16). IRF8 expression by tumor-associated macrophages (TAMs) was negatively associated with tumor stage and positively correlated with prognosis in ccRCC patients (17). As a component of IFN-stimulated gene factor 3 (ISGF3), IRF9 expression in ccRCC cells was negatively associated with tumor growth (18). The above results indicated that IRFs played a diverse regulatory role in the oncogenesis and progression of ccRCC. Cumulative evidences showed that carcinogenesis and progression of cancer was the consequence of the interaction of multiple genes and/or signal pathways (19). A single gene as biomarkers may be not sufficient to accurately predict prognosis and estimate immune status in ccRCC. Hence, we utilized all IRF family members to construct a novel risk model to provide new insights into predicting prognosis and promoting the individualized immunotherapy.

In our study, we classified ccRCC patients into different molecular subtypes based on IRFs and constructed a novel risk model. Moreover, we estimated the clinical performance of this risk model in terms of prognosis, immune microenvironment, response to immunotherapy and targeted drug sensitivity.

## Materials and methods

### Ethical statement

This study was approved by the Ethical Committee of Shandong Provincial Hospital Affiliated to Shandong First Medical University (SWYX: NO.2021-277). Written informed consent was obtained from all patients.

### Data preparation

Transcriptomic RNA (HTseq-FPKM) including 539 ccRCC tissues and 72 adjacent nontumor tissues with clinical information were acquired from The Cancer Genome Atlas (TCGA) database. The gene annotation of the gene transfer format (GTF, release 37, GRCh38.p13) file downloaded from GENECODE (http://gencodegenes.org) was used to annotate gene symbols. Somatic mutation data and copy number variation (CNV) data of TCGA-KIRC patients were downloaded from the USUC Xena (https://xena.ucsc.edu). In addition, three gene expression profiles of the GSE40435, GSE53757 and GSE66272 datasets with a total of 400 samples were downloaded from the Gene Expression Omnibus (GEO) database. After the batch effects were corrected using “sva” R package, the three datasets (GSE40435, GSE53757 and GSE66272) were merged into a single dataset. The single-cell RNA-sequencing (scRNA-seq) raw count files of the GSE156632 dataset was also obtained from the GEO database. The E-MTAB-1980 cohort comprising 101 ccRCC patients with clinical data was obtained from the EMBL-EBI database (https://www.ebi.ac.uk/).

### scRNA-seq data analysis

The 10× scRNA-seq data was converted to Seurat object using “Seurat” R package. The clusters with cells less than 3, cells that were detected less than 50 genes and cells that expressed more than 5% of mitochondrial genes were removed. Principal component analysis (PCA) was performed using the top 1500 most variable genes. The “FindNeighbors” and “FindClusters” functions were used for cell clustering analysis based on the top 15 principal components (PCs). The “FindAllMarkers” function was applied to identify marker genes of different cell clusters based on the threshold of FDR< 0.01 and |log_2_FC| > 1. Furthermore, cluster annotation was performed to recognize different cell type using “SingleR” package.

### Differential expression analysis of the IRF family members and gene-gene interaction network

The mRNA expression levels of the IRF family members in non-paired samples and paired samples were analyzed using Wilcoxon rank-sum test and Wilcoxon signed-rank test respectively based on the TCGA-KIRC dataset. The mRNA expression levels of the IRF family members between ccRCC samples and normal samples were validated based on the GEO dataset using the Wilcoxon signed-rank test. In addition, UALCAN (http://ualcan.path.uab.edu) was used to analyze the protein expression levels of IRF family members between ccRCC samples and normal samples according to data from the Clinical Proteomic Tumor Analysis Consortium (CPTAC). P< 0.05 was considered statistically significant. The correlation analysis of the IRF family members was performed on basis of their mRNA expression data from the TCGA-KIRC dataset.

### Prognostic values of the IRF family members

Kaplan-Meier (KM) survival curves were plotted to evaluate OS of the IRF family members in ccRCC based on the optimal cutoff value using “survival” R package. A receiver operating-characteristic (ROC) curve was plotted using the “pROC” R package, and the area under curve (AUC) was calculated to evaluate diagnostic capability of the IRF family members.

### Identification of molecular subtypes based on IRF family members

Based on the expression profiles of IRF family members, non-negative matrix factorization (NMF) with “brunet” method for 10 iterations was performed to cluster the TCGA-KIRC samples. The number of clusters was set as 2 to 10 and the average contour width of the common member matrix was determined using the “NMF” R package. The minimum number of each subset was set as 10. Then, the optimal number of clusters was determined according to cophenetic, dispersion and silhouette indexes. KM survival curve was used to explore the difference of OS between the different molecular subtypes. Besides, the difference in mRNA expression of IRF family members between the different molecular subtypes was analyzed. Differentially expressed genes (DEGs) between different molecular subtypes were identified using the “limma” R package with the threshold of FDR< 0.05 and |log_2_FC| > 1.

### Gene set variation analysis (GSVA) and functional enrichment analysis

GSVA was applied to explore the difference in biological pathways between the different molecular subtypes through “GSVA” R package. The gene sets of “c2.cp.kegg.v2022.1.Hs.symbols.gmt” were obtained from the MSigDB database. Gene Ontology (GO) and Kyoto Encyclopedia of Genes and Genomes (KEGG) pathway enrichment analyses were performed with the “clusterprofiler”, “org.Hs.eg.db”, “enrichplot” and “circlize” R packages. The enrichment categories were considered as statistically significant if a false discovery rate (FDR)< 0.05.

### Construction and validation of an IRFs-related prognostic model

Subsequently, the prognostic-related DEGs were identified by univariate Cox regression analysis based on the TCGA-KIRC cohort (*p*<0.01). To avoid the overfitting risk, least absolute shrinkage and selection operator (LASSO) Cox regression analysis was performed to narrow down the candidate genes using the “glmnet” R package. Finally, multivariate Cox regression analysis was conducted to determine the target genes for constructing an IRFs-related prognostic model. The risk score was calculated as follows: risk score = $\sum_{\text{i}=1}^{\text{n}}\text{Expi}*\text{coefi}$ (where n, Expi and coefi represent the number of genes, the expression of each gene, and risk coefficient of each gene, respectively). According to the median value of the risk score, patients were divided into the high-risk and low-risk groups. Survival analysis was conducted to explore differences in the OS between the high-risk and low-risk groups. Additionally, time-dependent ROC curve using “timeROC” R package was plotted, and the 1-, 3- and 5-year AUCs were calculated to evaluate the sensitivity and specificity of the prognostic model. PCA and t-distributed stochastic neighbor embedding (*t*-SNE) were performed to explore the distribution of the two risk groups. The E-MTAB-1980 cohort was used as an external independent cohort to validate the prognostic model.

Furthermore, we evaluated the relationships between the risk score and clinical characteristics. Univariate and multivariate Cox regression analyses were used to evaluate whether the risk score could serve as an independent prognostic biomarker. A nomogram combining the risk score and clinical characteristics (age, gender and stage) was constructed to predict the 1-, 3- and 5-year OS of ccRCC patients. To evaluate the predictive accuracy of the nomogram, the calibration curve and concordance index (C-index) curve were plotted. Decision curve analysis (DCA) was performed to evaluate the clinical utility and net benefit of the nomogram.

### Evaluation of immune characteristics

To explore the immune status in ccRCC, the ESTIMATE algorithm was used to calculate the stromal score and immune score of each sample. The abundance of 22 immune cells was estimated using the CIBERSORT algorithm. The infiltration levels of 16 immune cells and activity scores of 13 immune-related pathways were calculated by the single sample gene set enrichment analysis (ssGSEA). The cancer immunity cycle including seven steps could reflect anticancer immune response in tumor microenvironment (TME) (20). Therefore, we compared the differences in the immune activity scores of the seven steps between the high-risk and low-risk groups based on the Tracking Tumor Immunophenotype (TIP; http://biocc.hrbmu.edu.cn/TIP/index.jsp) database. Furthermore, tumor mutation burden (TMB) of each patient in the TCGA-KIRC cohort was calculated. The difference in TMB between the high-risk and low-risk groups was compared, and the correlation between the risk score and TMB was also analyzed.

### Assessment of immunotherapy response

To evaluate the immunotherapy response between the high-risk and low-risk groups, the tumor immune dysfunction and exclusion (TIDE; http://tide.dfci.atherard.edu/) was used to calculate the TIDE score of each patient according to myeloid-derived suppressor cell (MDSC), macrophage M2, T cell Dysfucntion and Exclusion (21). Moreover, the T-cell inflammatory signature (TIS) score was calculated based on the mean value of a log2-scaled normalized expression of 18 signature genes (22). The ROC curve was conducted to compare the predictive ability of risk model, TIDE and TIS using “timeROC” R package.

### Drug sensitivity analysis

Based on the Genomics of Drug Sensitivity in Cancer (GDSC; https://www.cancerrxgene.org/) database, the half-maximal inhibitory concentration (IC50) of chemotherapeutic drugs was estimated using the “oncoPredict” R package. Thereafter, the difference in IC50 between the high-risk and low-risk groups was analyzed by Wilcoxon signed-rank test.

### RNA extraction and quantitative real-time polymerase chain reaction (qRT-PCR)

20 pairs of ccRCC tissues and adjacent normal tissues were collected and stored at -80°C for qRT-PCR. Total RNA was extracted from 20 pairs of ccRCC tissues and adjacent normal tissues using TRIzol (TaKaRa, Japan) in accordance with the manufacturer’s instructions. The T100 Thermal Cycler (Bio-Rad, USA) was used to reverse-transcribe RNA into cDNA. qPCR reactions were performed using Fast Start Universal SYBR Green Master (Roche, Switzerland) in the LightCycler 480 (Roche, Switzerland). The qPCR conditions were as follows: (1) 30 s at 95°C; (2) 5 s at 95°C, and 30 s at 60°C for 45 cycles; and (3) melt curve analysis. The sequences of primers are shown in **Supplementary Table S1**. The relative mRNA expression levels of IRF family members were calculated by the 2^-△△CT^ method.

### Immunohistochemistry (IHC)

In addition, ccRCC tissues and adjacent normal tissues were fixed in formalin and embedded in paraffin for IHC analysis. Tissue sections (4 μm in thickness) were cut from the clinical samples (ccRCC tissues and normal tissues). The sections were placed in an oven at 72°C for two hours to prevent the tissues from falling out. Then, the sections were deparaffinized with xylene, rehydrated with ethanol and placed in sodium citrate buffer in a pressure cooker for antigen retrieval. Next, the sections were immersed into 3% hydrogen peroxide solution for 4 min at room temperature to inactivate endogenous peroxidase, and then they were rinsed in phosphate-buffered saline (PBS). The sections were incubated with primary antibodies against IRF1 (Abclonal, Wuhan, China), IRF2 (Abclonal), IRF3 (Abclonal), IRF4 (Abcam, Cambridge, UK), IRF5 (Abclonal), IRF6 (HUABIO, Hangzhou, China), IRF7 (Proteintech, Wuhan, China), IRF8 (Abcam) and IRF9 (Proteintech) at 4°C overnight. Then, the sections were incubated with secondary antibodies at room temperature for 40 min. Subsequently, the sections were stained with 3,3’-diaminobenzidine (DAB) and counterstained with hematoxylin. We examined three fields of view (200x) selected randomly from each section. The average optical density (AOD) value of each image was measured by Image J software, and the difference in AOD value between ccRCC tissues and normal tissues was compared using Wilcoxon test.

## Results

### Multi-omics landscape of IRF family members in ccRCC

**Figure 1:**
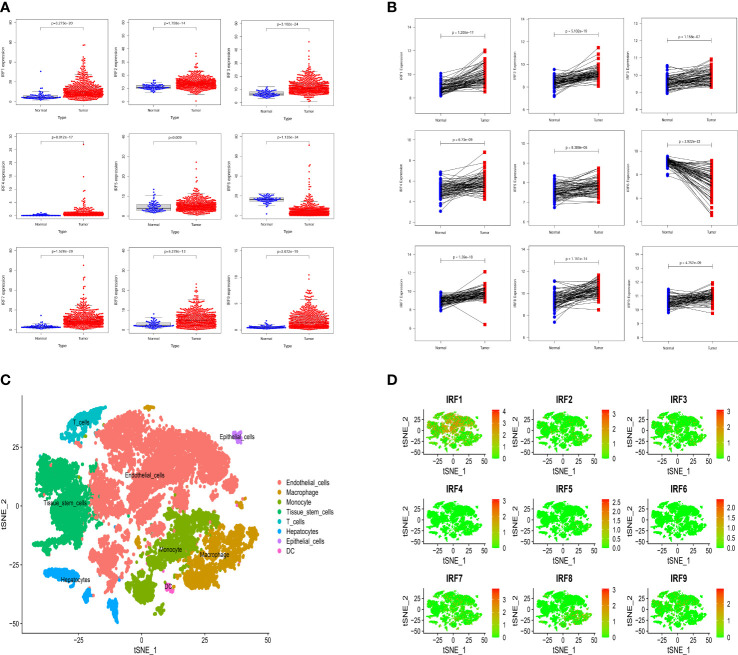
The expression levels of the IRF family members between ccRCC samples and normal samples. **(A)** The mRNA expression levels of the IRF family members in the TCGA-KIRC dataset. **(B)** The mRNA expression levels of the IRF family members in the GEO dataset. **(C)** The cell types were identified by single-cell RNA-sequencing analysis. **(D)** The expression levels of the IRF family members in different types of cell clusters.

**Figure 2:**
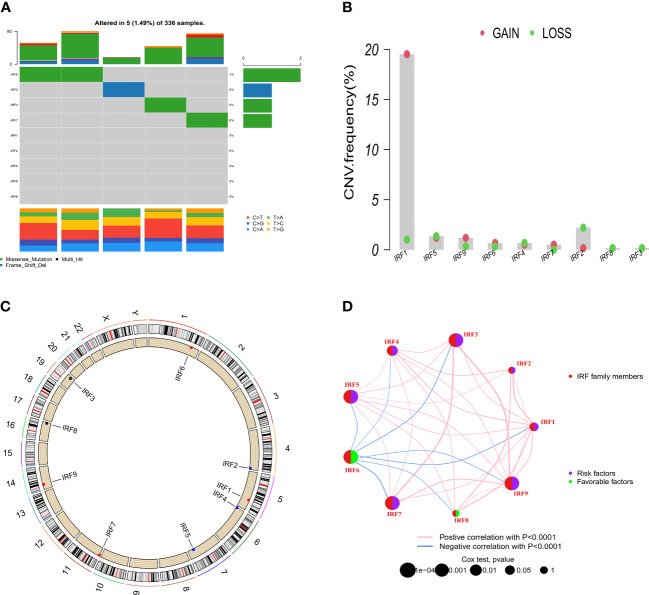
Somatic mutation and CNVs frequencies of the IRF family members in ccRCC. **(A)** Mutation frequency of the IRF family members in 336 patients with ccRCC. **(B)** CNVs of the IRF family members. **(C)** Locations of the CNV alterations of the IRF family members on 23 chromosomes. **(D)** Correlations and prognosis of the IRF family members in ccRCC patients.

Based on the TCGA-KIRC dataset, the mRNA expression levels of IRF1/2/3/4/5/7/8/9 in 539 ccRCC samples were significantly higher than those in 72 normal samples, whereas the mRNA expression level of IRF6 in 539 ccRCC samples was significantly lower than that in 72 normal samples (**Figure 1A**). Moreover, the mRNA expression trends of the IRF family members, except for IRF5, in paired samples were consistent with those in non-paired samples (**Supplementary Figure S1**). The result in the GEO dataset showed that the expression levels of IRF1/2/3/4/5/7/8/9 in ccRCC samples were significantly upregulated compared with those in the normal samples, whereas the expression level of IRF6 in ccRCC samples was significantly downregulated compared with that in the normal samples (**Figure 1B**). On basis of the scRNA-seq data, we further validated the expression of the IRF family members in different types of cells in the TME. Eight cell clusters, namely endothelial cells, macrophage, monocyte, tissue stem cells, T cells, hepatocytes, epithelial cells and DC, were identified (**Figure 1C**) and the expression levels of the IRF family members in different types of cell clusters were shown in **Figure 1D**. Furthermore, we found that the protein levels of IRF2/3/4/7/8/9 in ccRCC samples were higher than those in the normal samples, while the protein level of IRF6 in ccRCC samples was lower than that in the normal samples (**Supplementary Figure S2**). The incidence of somatic mutation and CNVs of IRFs were also estimated. Among the 336 samples, only 5 samples (1.49%) had mutations in IRF family members (**Figure 2A**). We also found IRF1 and IRF9 had copy number amplification, while IRF2 had copy number deletion (**Figure 2B**). The location of CNV alterations of IRF family members on the chromosomes were shown in **Figure 2C**. A correlation network of IRF family members was constructed to show the whole landscapes of their interactions and prognostic values (**Figure 2D**). KM survival curves showed that the high expression of IRF1 (p = 0.049), IRF3 (p< 0.001), IRF4 (p< 0.001), IRF5 (p< 0.001), IRF7 (p< 0.001) and IRF9 (p< 0.001), and the low expression of IRF2 (p = 0.049) and IRF6 (p< 0.001) were significantly associated with poor OS (**Supplementary Figure S3**). We also found that IRF1, IRF3, IRF4, IRF5 and IRF7 were significantly higher in tumor stage III/IV or grade 3/4 compared with tumor stage I/II or grade 1/2, whereas the expression level of IRF6 was lower in tumor stage III/IV or grade 3/4 (**Supplementary Figure S4**). These findings suggested that IRF family members might serve an important role in the oncogenesis and progression of ccRCC. Subsequently, multivariate Cox regression analysis identified that IRF9 (HR: 1.174; 95% CI: 1.051-1.311; *p* = 0.004) was an independent prognostic risk factor (**Supplementary Figures S5A, B**). ROC curve revealed that IRF9 (AUC = 0.826) had good diagnostic value for ccRCC (**Supplementary Figure S5C**). Nonetheless, time-dependent ROC curves indicated that *IRF9* (1-, 3-, 5-year AUC: 0.581, 0.581 and 0.656, respectively) had low predictive capability for the OS (**Supplementary Figure S5D**).

### Validation of the IRF family members by qRT-PCR and IHC

**Figure 3:**
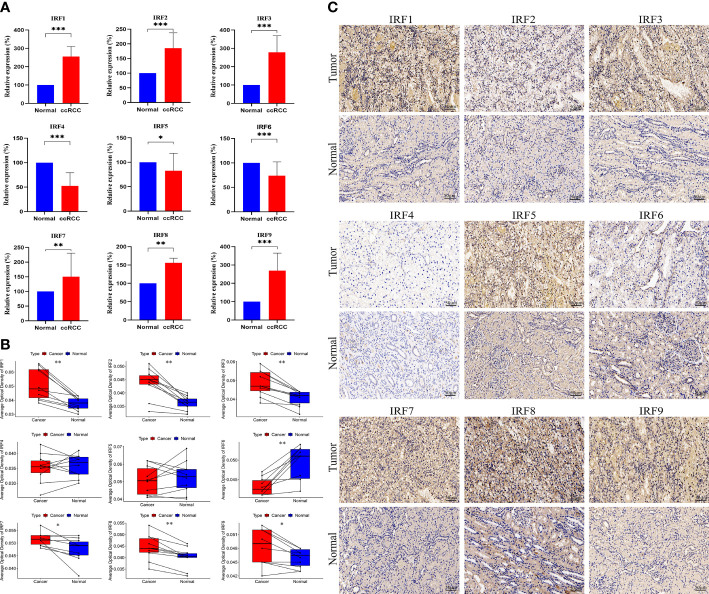
QRT-PCR and IHC analyses of the IRF family members. **(A)** The relative mRNA expression levels of the IRF family members between ccRCC and normal tissues were validated by qRT-PCR. **(B)** The AOD values of the IRF family members between ccRCC and normal tissues were compared. **(C)** Representative IHC staining of the IRF family members between ccRCC and normal tissues were shown. * *p*<0.05, ** *p*<0.01, *** *p*<0.001.

We performed qRT-PCR to examine the mRNA expression levels of the IRF family members in clinical specimens. As shown in **Figure 3A**, the relative mRNA expression levels of IRF1/2/3/7/8/9 in ccRCC tissues were significantly higher than those in the normal tissues, whereas the relative mRNA expression levels of IRF4/5/6 in ccRCC tissues were significantly lower than those in the normal tissues. The mRNA expression trends of the IRF family members, except for IRF4/5, were consistent with the results of the above bioinformatics analysis. Meanwhile, IHC was conducted to validate the protein expression levels of the IRF family members between ccRCC tissues and normal tissues (**Figures 3B, C**). The result revealed that the protein levels of IRF1/2/3/7/8/9 in ccRCC tissues were higher than those in the normal tissues, while the protein level of IRF6 in ccRCC tissues was lower than that in the normal tissues.

### Identification of IRFs-related molecular subtypes

**Figure 4:**
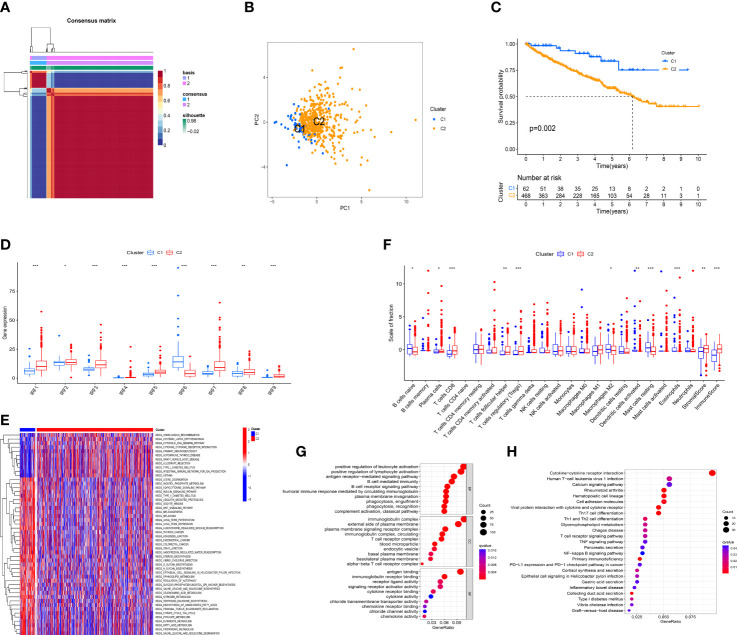
Identification of IRFs-related molecular subtypes. **(A)** Consensus map of NMF clustering (k = 2). **(B)** PCA plot of the expression profiling of IRFs. **(C)** KM analysis of OS between the two molecular subtypes. **(D)** The differences in the expression levels of IRF family members between the two molecular subtypes. **(E)** Heatmap of biological pathways between the two molecular subtypes. Activated and inhibited pathways are colored by red and blue, respectively. **(F)** The differences in immune score, stromal score and immune infiltrating cells between the two molecular subtypes. **(G)** GO enrichment analysis of DEGs between the two molecular subtypes. **(H)** KEGG pathway enrichment analysis of DEGs between the two molecular subtypes. * *p*<0.05, ** *p*<0.01, *** *p*<0.001.

According to the expression profile of IRF family members, unsupervised NMF algorithm was performed to identify novel IRF-related molecular subtypes in ccRCC. The optimal number of the clusters was identified as two (k =2). Consequently, the TCGA-KIRC cohort was divided into C1 (n = 62) and C2 (n = 468) subtypes (**Figure 4A**). PCA showed diverse clustering of the two molecular subtypes (**Figure 4B**). Survival analysis showed that the patients in C2 subtype had a worse OS than those in C1 subtype (**Figure 4C**). The distribution of clinical characteristics between the two molecular subtypes was illustrated in **Supplementary Figure S6**. As expected, all IRF family members showed significant differences between the two molecular subtypes (**Figure 4D**). In addition, GSVA enrichment analysis showed that C1 subtype was enriched in Wnt signaling pathway, thyroid cancer, colorectal cancer, regulation of autophagy and fatty acid metabolism, while C2 subtype was enriched in cytosolic DNA-sensing pathway, cytokine-cytokine receptor interaction and primary immunodeficiency (**Figure 4E**). Simultaneously, we estimated the differences in immune score, stromal score and immune infiltrating cells between the two molecular subtypes. The result revealed that immune score and stromal score in C2 subtype were significantly higher than those in C1 subtype. Additionally, naïve B cells, M2 macrophages, activated dendritic cells, resting mast cells and eosinophils were remarkably higher in C1 subtype, whereas plasma cells, CD8 T cells, T follicular helper cells (Tfh) and T regulatory cells (Tregs) were significantly higher in C2 subtype (**Figure 4F**). These results all indicated that there was a significant difference in immune microenvironment between the two molecular subtypes.

To further explore the heterogeneity between the two molecular subtypes, 1425 DEGs were identified with the threshold of FDR< 0.05 and |log_2_FC| > 1. GO and KEGG pathway enrichment analyses for these DEGs were performed. GO analysis revealed that these DEGs were mainly concentrated on biological processes related to immune regulatory processes, such as positive regulation of lymphocyte activation, B cell mediated immunity, T cell receptor complex, and chemokine activity (**Figure 4G**). Moreover, KEGG pathway analysis showed that these DEGs were mainly enriched in cytokine-cytokine receptor interaction, Th17 cell differentiation, Th1 and Th2 cell differentiation, T cell receptor signaling pathway, TNF signaling pathway, NF-κB signaling pathway, and PD-L1 expression and PD-1 checkpoint pathway in cancer (**Figure 4H**). Hence, it is supposed that IRFs might be closely involved in regulating immune cells and immune responses in the TME of ccRCC.

### Construction and validation of an IRFs-related prognostic model

**Figure 5:**
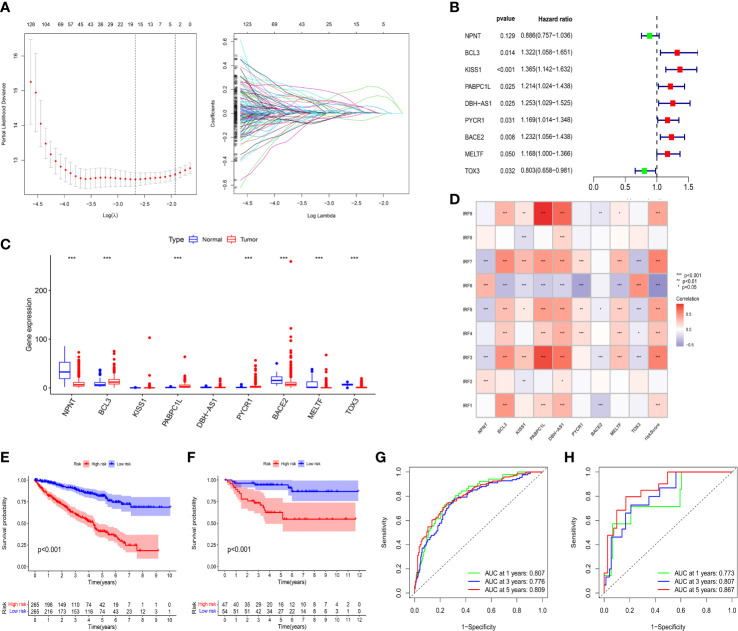
Construction and validation of an IRFs-related prognostic model. **(A)** The LASSO Cox regression analysis was performed to filter out the candidate genes. **(B)** 9 genes were retained to construct a prognostic model using the multivariate Cox regression analysis. **(C)** The mRNA expression levels of the nine genes between ccRCC samples and normal samples in the TCGA-KIRC dataset. **(D)** Correlations between IRF family members and risk score. **(E, F)** KM curves of OS between the low- and high-risk groups in TCGA-KIRC and E-MTAB-1980 datasets. **(G, H)** ROC curves of the IRFs-related prognostic model in predicting the 1-, 3- and 5-year OS in the TCGA-KIRC and E-MTAB-1980 datasets. * *p*<0.05, ** *p*<0.01, *** *p*<0.001.

By performing univariate Cox regression analysis, 421 prognostic-related DEGs were identified based on TCGA-KIRC cohort (**Supplementary Table S2**). To avoid overfitting risk and narrow down the range of candidate genes, LASSO Cox regression analysis was conducted to further filter out 19 candidate genes (**Figure 5A**). Finally, 9 genes (NPNT, BCL3, KISS1, PABPC1L, DBH-AS1, PYCR1, BACE2, MELTF, and TOX3) were retained to construct an IRFs-related prognostic model using the multivariate Cox regression analysis (**Figure 5B**). The risk score of each patient in both TCGA-KIRC and E-MATB-1980 cohorts was calculated using the following formula: risk score = expression of NPNT*(-0.12142) + expression of BCL3*(0.278869) + expression of KISS1*(0.3112) + expression of PABPC1L*(0.193679) + expression of DBH-AS1*(0.225393) + expression of PYCR1*(0.156245) + expression of BACE2*(0.208868) + expression of MELTF*(0.155669) + expression of TOX3*(-0.21914). Then, we examined the expression levels of the nine genes based on the TCGA-KIRC cohort and found that the expression levels of BCL3, PABPC1L and PYCR1 in ccRCC samples were higher than those in normal samples, while the expression levels of NPNT, BACE2, MELTF and TOX3 in ccRCC samples were lower than those in normal samples (**Figure 5C**).

Patients were stratified into low-risk and high-risk groups according to the median value of risk score. PCA and *t*-SNE revealed that patients in the two risk groups were distributed in diverse directions in both TCGA-KIRC and E-MTAB-1980 cohorts (**Supplementary Figures S7A–D**). Additionally, there were remarkably differences in expression levels of IRF1/3/4/5/6/7/9 between the high-risk and low-risk groups (**Supplementary Figure S7E**). Meanwhile, we found that IRF family members were positively or negatively correlated with risk score and target genes in the risk model (**Figure 5D**). Survival analysis indicated that the patients in the low-risk group had a better OS than those in the high-risk group whether in the TCGA-KIRC (**Figure 5E**) or E-MTAB-1980 cohorts (**Figure 5F**). Furthermore, time-dependent ROC curves were plotted to explore the predictive capability of the prognostic model. The 1-, 3- and 5-year AUCs in TCGA-KIRC cohort were 0.807, 0.776 and 0.809, respectively (**Figure 5G**). Similarly, the 1-, 3- and 5-year AUCs in E-MTAB-1980 cohort were 0.773, 0.807 and 0.867, respectively (**Figure 5H**).

### Correlation between risk score and clinical characteristics

**Figure 6:**
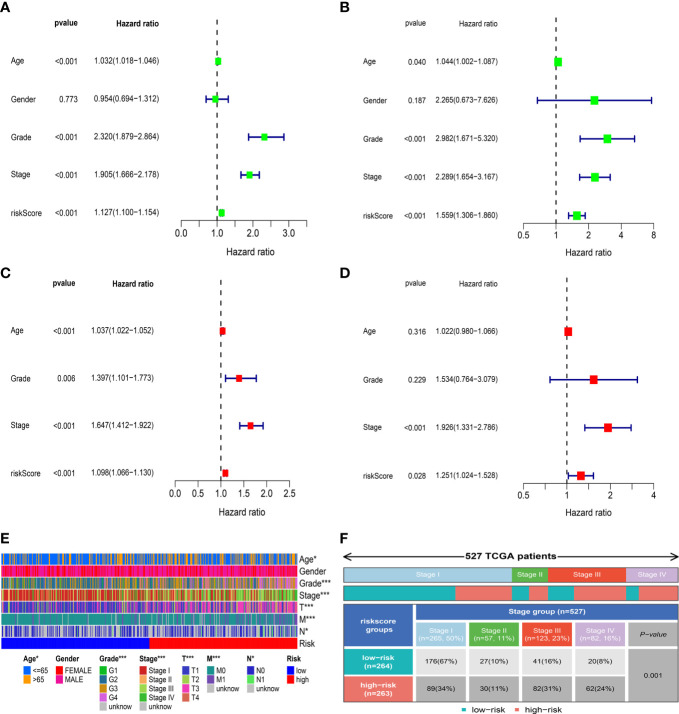
Correlation between risk score and clinical characteristics. **(A, C)** Univariate and multivariate Cox regression analyses showed that risk score was an independent prognostic indicator in the TCGA-KIRC dataset. **(B, D)** Univariate and multivariate Cox regression analyses showed that risk score was an independent prognostic indicator in the E-MTAB-1980 dataset. **(E)** Differences in clinical characteristics between the low- and high-risk groups in the TCGA-KIRC dataset. **(F)** Distribution of tumor stages between the low- and high-risk groups. * p<0.05, *** p<0.001.

**Figure 7:**
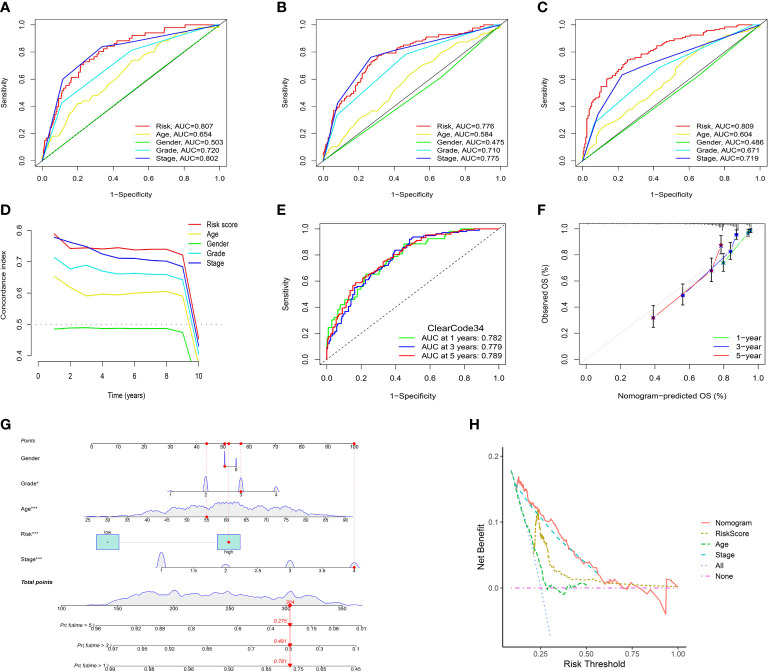
Assessment of the IRFs-related prognostic model and construction of a nomogram to predict the OS. **(A-C)** ROC curves of the nomogram in predicting the 1-,3- and 5-year OS in the TCGA-KIRC dataset. **(D)** C-indexes of the risk score and clinical characteristics. **(E)** ROC curves of the ClearCode34 model in predicting the 1-, 3- and 5-year OS. **(F)** The calibration curve of the nomogram in predicting the 1-, 3- and 5-year OS. **(G)** Construction of a nomogram based on age, gender, stage and risk score. **(H)** DCA curve of the nomogram. * p<0.05, *** p<0.001

To evaluate the independent prognostic value of the IRFs-related prognostic model, univariate and multivariate Cox regression analyses were performed in both TGCA-KIRC and E-MTAB-1980 cohorts. Univariate Cox regression analysis revealed that the risk score in both the TCGA-KIRC (**Figure 6A**; HR = 1.127, 95% CI:1.100-1.154, *p*< 0.001) and E-MTAB-1980 (**Figure 6B**; HR = 1.559, 95% CI:1.306-1.860, *p*< 0.001) cohorts was significantly associated with OS. After adjusting for confounding factors by multivariate Cox regression analysis, the risk score was confirmed to be an independent prognostic indicator in ccRCC patients (TCGA-KIRC: **Figure 6C**, HR = 1.098, 95% CI: 1.066-1.130, *p*< 0.001; E-MTAB-1980: **Figure 6D**, HR = 1.251, 95% CI: 1.024-1.528, *p* = 0.028). According to the TCGA-KIRC cohort, the relationships between clinical characteristics and risk score were explored, and the result revealed a significant difference in age, grade and TNM stage (**Figure 6E**). Furthermore, **Figure 6F** showed that there were more ccRCC patients with stage I-II in the low-risk group, but there were more ccRCC patients with stage III-IV in the high-risk group (*p*< 0.001). Besides, the C-index and ROC curve were conducted to evaluate the predictive performance of the risk model. We found that the C-index of the risk score was higher than those of other clinical characteristics (**Figure 7D**), suggesting the risk score could better predict the prognosis of ccRCC patients. Similarly, ROC curves also revealed that the AUC of the risk score was higher than those of other clinical characteristics, indicating that the risk score had higher sensitivity and specificity in predicting prognosis of ccRCC patients (**Figures 7A–C**). As reported, the robust predictive power of a ClearCode34 model has been validated in clinical cohorts (23, 24). We performed the 1-, 3-, and 5-year ROC curves of the ClearCode34 model (**Figure 7E**), and found that the 1-, 3-, and 5-year AUCs of IRFs-related risk model were higher than those of the ClearCode34 model, indicating that IRFs-related risk model was superior to the ClearCode34 model in predicting the prognosis of ccRCC.

### Construction and evaluation of the prognostic nomogram

A nomogram scoring system comprising age, gender, stage and risk score was constructed to predict the 1-, 3- and 5-year OS of ccRCC patients based on the TCGA-KIRC cohort (**Figure 7G**). The excellent consistency of the calibration curve suggested that the nomogram had a high accuracy to predict the 1-, 3- and 5-year OS in ccRCC patients (**Figure 7F**). ROC curves revealed that the 1-, 3- and 5-year AUCs of the nomogram were 0.866, 0.822 and 0.793, indicating the nomogram showed satisfactory predictive ability, which was superior to other clinical characteristics (**Supplementary Figures S8A–C**). Furthermore, DCA revealed that the nomogram had better net benefit than other clinical characteristics (**Figure 7H**).

### Evaluation of immune characteristics and immunotherapeutic response

**Figure 8:**
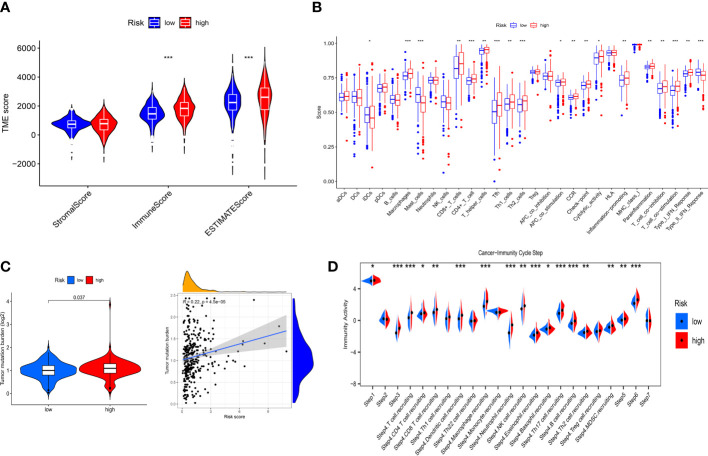
Immune landscape between the low- and high-risk groups. **(A)** Differences in the stromal score, immune score and ESTIMATE score. **(B)** Differences in the 16 immune cells and 13 immune-related pathways between the low- and high-risk groups. **(C)** Correlation between TMB and risk score. **(D)** Differences in the immune activity score of cancer-immunity cycle steps between the low- and high-risk groups. * p<0.05, ** p<0.01, *** p<0.001.

To further explore the correlation between immune landscape and the risk score, the ESTIMATE algorithm was used to calculate the immune score, stromal score and ESTIMATE score. The high-risk group had a higher ESTIMATE score and immune score than the low-risk group (**Figure 8A**), indicating that ccRCC patients in the high-risk group might present more active immune status. Subsequently, the ssGSEA was used to explore the infiltration levels of 16 immune cells and activity scores of 13 immune-related pathways between the two risk groups. We found that the high-risk group had higher infiltration levels of CD8^+^ T cell, CD4^+^ T cell, macrophages, T helper (Th) cells, Tfh, Type 1 T helper (Th1) cells and Type 2 T helper (Th2) cells, whereas the low-risk group had higher infiltration levels of immature dendritic cells (iDCs) and mast cells (**Figure 8B**). Moreover, the activity scores of APC co-stimulation, CCR, check point, cytolytic activity, inflammation promoting, parainflammation, T cell co-inhibition, T cell co-stimulation and type I IFN response were higher in the high-risk group, whereas the activity score of type II IFN response was lower in the high-risk group (**Figure 8B**). Thorsson et al. (25) have identified six cancer immune subtypes (IS) including IS1 (wound healing), IS2 (IFN-γ dominant), IS3 (inflammatory), IS4 (lymphocyte depleted), IS5 (immunologically quiet), and IS6 (TGF-β dominant). As shown in **Supplementary Figure 8D**, there was significant difference in immune subtypes between the two risk groups and there were more patients with IS3 immune subtype in both the high-risk and low-risk groups (*p*< 0.001). To further explore the activity of immune cells in ccRCC, we calculated the immune activity score of each step based on TIP database. We discovered that the immune activity scores of most steps in the high-risk group were remarkably higher than those in the low-risk group (**Figure 8D**). Furthermore, we found that the high-risk group presented a more extensive TMB level than the low-risk group, and TMB level was positively associated with the risk score (**Figure 8C**). However, clinical researches have demonstrated that TMB could not predict the therapeutic response to ICIs in ccRCC (26, 27).

**Figure 9:**
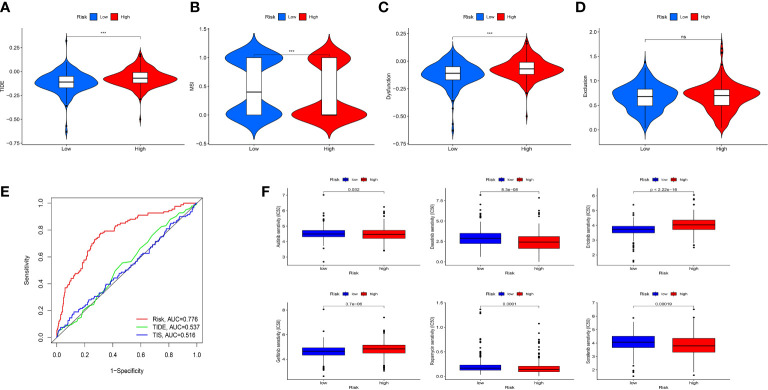
Evaluation the value of the IRFs-related prognostic model in immunotherapy and drug sensitivity. **(A-D)** Differences in TIDE, MSI, T cell dysfunction and T cell exclusion between the low- and high-risk groups. **(E)** ROC curve of IRFs-related prognostic model, TIDE and TIS in predicting the OS. **(F)** Correlation between risk score and drug sensitivity. *** *p*<0.001. ns, no significance.

To evaluate the value of the risk model in immunotherapy, the relationships between risk score and TIDE, T-cell dysfunction, T-cell exclusion score and MSI score were explored. The result showed that TIDE score in the high-risk group was higher than that in the low-risk group, indicating patients in the low-risk group were more likely to benefit from ICIs therapy than those in the high-risk group (**Figure 9A**). Besides, we found that high-risk group showed a higher T-cell dysfunction and lower MSI score than low-risk group (**Figures 9B–D**). Meanwhile, ROC curve showed that the AUC of IRF-related risk model was remarkably higher than that of TIS and TIDE (**Figure 9E**), which suggested that the risk model displayed better predictive value for prognosis in ccRCC than TIS and TIDE.

### Drug sensitivity analysis

To explore the correlation between the risk score and response to targeted drugs of ccRCC, we compared the differences in IC50 of these drugs between the high-risk and low-risk groups. We observed that the IC50 of axitinib, sorafenib, dasatinib, and rapamycin in the high-risk group were lower than those in the low-risk group, while the IC50 of erlotinib and gefitinib in the high-risk group were higher than those in the low-risk group (**Figure 9F**). Thus, we proposed that IRFs-related risk model could serve as a potential predictive factor for the sensitivity of targeted drugs.

## Discussion

ccRCC is a heterogeneous tumor with high infiltration levels of immune cells, high aggressiveness and poor prognosis (28, 29). Intratumor heterogeneity in ccRCC is considered to be related to patterns of metastatic spread and prognosis, which makes it complex to predict prognosis and determine the appropriate therapeutic strategies (30). Moreover, the heterogeneity of tumor microenvironment (TME) might be responsible for the distinct therapeutic responses to ICIs in ccRCC patients (10). Cumulative evidences showed that IRFs participated in regulating immune cells and immune-related pathways in cancers (11), which suggested that IRFs might play an essential role in TME. Hence, identifying IRFs-related risk model is naturally significant to stratify ccRCC patient heterogeneity, predict prognosis and develop the individualized immunotherapeutic strategies.

Herein, multi-omic analysis of IRF family members in ccRCC indicated that IRFs might play an important role in oncogenesis and progression of ccRCC. Subsequently, the NMF algorithm was used to classify ccRCC patients into two distinct molecular subtypes based on the expression profile of IRF family members. We discovered that the patients in C2 subtype showed a worse OS than those in C1 subtype. In addition, there were differences in immune score, stromal score and abundance of various immune cells between the two molecular subtypes. Furthermore, GO and KEGG pathway enrichment analyses showed enrichment of immune-related pathways, such as positive regulation of lymphocyte activation, B cell mediated immunity, chemokine activity, cytokine-cytokine receptor interaction, Th17 cell differentiation, Th1 and Th2 cell differentiation, T cell receptor signaling pathway, TNF signaling pathway, NF-κB signaling pathway, and PD-L1 expression and PD-1 checkpoint pathway in cancer. It was evidenced that regulatory B cells could attenuate antitumor immune responses by suppressing the T-cell immune response (31). Cytokines and chemokines were found to play a crucial role in cancer-related inflammation and immune escape (32). Qu et al. revealed that the TNF-α/TNFR2 pathway was activated to enhance the immunosuppressive phenotype and function of Tregs in TME of gastric cancer (33). Overexpression of miR-210-3p could promote epithelial-mesenchymal transition, invasion, migration and bone metastasis in prostate cancer by activating NF-κB signaling pathway (34). IFNγ could promote tumor immune escape by regulating the PD-L1 expression *via* the JAK/STAT and PI3K-AKT signaling pathways (35). Taken together, it is reasonable to propose that IRFs were significantly involved in oncogenesis and progression of ccRCC through regulating immune responses and/or immune-related pathways.

We identified 9 target genes (NPNT, BCL3, KISS1, PABPC1L, DBH-AS1, PYCR1, BACE2, MELTF, and TOX3) to construct an effective and robust prognostic model in the TCGA-KIRC cohort, and validated the performance of the prognostic model in the E-MTAB-1980 cohort. Some target genes in the prognostic model have been explored in ccRCC. For instance, Braga et al. revealed that p50 together with Bcl-3 played an important role in the regulation of gene transcription in RCC (36). The invasiveness and colonized ability in RCC cells were inhibited through the activation of KISS1/KISS1R signaling by honokiol (37). Bioinformatic analysis showed that PYCR1 may contribute to create an immunosuppressive microenvironment in the TME, and thus it could be as potential target in the immunotherapy for ccRCC (38). Jiang et al. found that TOX3 overexpression could inhibit the epithelial-mesenchymal transition (EMT) to reduce cell migration and invasion *via* transcriptionally repressing SNAI1 and SNAI2 in ccRCC cells (39). However, the other genes were revealed for the first time, which remains to be further explored in ccRCC. Survival analysis demonstrated that patients in the low-risk group had a remarkably better prognosis. Multivariate Cox regression analysis indicated that the risk model was an independent prognostic indicator. Moreover, IRFs-related risk model was superior to the ClearCode34 model in predicting the prognosis. To improve the predictive performance of the risk model, we then constructed a nomogram comprising risk score and clinical characteristics to accurately predict prognosis for ccRCC, which was superior to conventional clinical characteristics.

The ccRCC is reported to be one of the cancers with highly immune infiltration by pan-cancer analysis (40). In the TME, immune cells serve a critical role in cancer growth, invasion, migration and regulating anticancer immunity (41). Recent studies revealed that high infiltration of CD8^+^ T cells was observed in ccRCC, which was closely correlated with the poor prognosis (42, 43). In addition, overexpression of immune escape markers and enhanced the infiltration levels of immunosuppressive cells were related to the high infiltration of CD8^+^ T cells in ccRCC (44, 45). Similarly, it was evidenced that the infiltration of Tregs and Tfh in ccRCC indicated a poor prognosis (46, 47). Moreover, high infiltration of tumor-associated macrophages (TAMs) correlated with the poor prognosis and tumor metastasis of cancers (48, 49). Şenbabaoğlu et al. found that the infiltration of mast cells was significantly negatively associated with OS and progression-free survival (PFS) in ccRCC (46). Consistent with these studies, we discovered that high infiltration of CD8^+^ T cells, macrophages and Tfh but low infiltration of mast cells in the high-risk group were associated with a worse prognosis. Interestingly, we also found higher activity scores of inflammation promoting and type I IFN response were in the high-risk group. Type I IFNs could be induced by IRF1/3/5/7/8 through Toll-like receptor (TLR) signaling and cGAS-STING pathways (50, 51). Meanwhile, evidences showed that type I IFNs offered proinflammatory mediators that contribute to tumor progression and increased negative regulatory cells and factors to promote immune escape (52). However, patients in the high-risk group presented lower activity of type II IFN response and showed higher expression of IRF1, which seemed to contradict the theory that activation of IFN-γ can induce IRF1 expression (51). In fact, IRF1 transcription can be driven not only by IFN-γ but also by proinflammatory NF-κB (51, 53). Previous studies showed that the excessive activation of NF-κB was closely associated with increased resistance to chemotherapy or cytokine therapy and a worse prognosis in ccRCC patients (54). Combined with KEGG enrichment analysis showing that NF-κB signaling pathway had a close relationship with IRFs-related molecular subtypes, it is supposed that NF-κB rather than IFN-γ played a major role in the regulation of IRF1 expression in ccRCC patients with high-risk. Additionally, IRF4 expression was excessively elevated in exhausted T cells that reduced IFN-γ production, which was in accordance with our results (55). To summarize, the reciprocal crosstalk between IRFs and IFNs might be responsible for the immune evasion and poor outcome in ccRCC patients. Furthermore, we also found that patients in high-risk group had higher immune scores and ESTIMATE scores. In accordance with the above findings, we believed that IRFs-related risk model could be an effective indicator for predicting prognosis and reflecting immune cells infiltration in the TME of ccRCC.

In recent years, ICIs have been widely used in immunotherapy for ccRCC. However, ccRCC patients exhibited diverse therapeutic responses to ICIs, which might be due to the heterogeneity of TME (10). Thus, it is extremely important to predict which patients can respond to ICIs. TIDE scores were associated with the potential of anticancer immune evasion, thereby predicting the therapeutic response to anti-PD1 and anti-CTLA4 (21). Moreover, high MSI showed a better response to immunotherapy (56). Our analysis showed that patients in low-risk group had lower TIDE score and T-cell dysfunction but a higher MSI than those in high-risk group, indicating that patients in low-risk group had a better response to ICIs. At the moment the combination of immunotherapy with targeted therapy have been deemed to be the first-line treatment for advanced ccRCC (57, 58). Thus, we next explored the response to targeted drugs in different risk groups. As expected, patients in different risk groups showed diverse drug sensitivity to axitinib, sorafenib, gefitinib, erlotinib, dasatinib and rapamycin. To summarize, the IRF-related risk model may be a valid tool to evaluate the response to both immunotherapy and targeted therapy, which can promote the development of personalized therapy for ccRCC patients.

In conclusion, we explored the different molecular subtypes of ccRCC based on IRF family members and evaluated the clinical prognosis, immune cell infiltration and signaling pathways of different molecular subtypes. Furthermore, we developed a robust and effective risk model to predict prognosis and responses to ICIs and targeted drugs and reflect the TME characteristics in ccRCC. These findings might provide new insights into personalized and precise therapeutic strategies. However, there were several limitations in our study. First, the public TCGA-KIRC and E-MTAB-1980 retrospective cohorts were used to construct and validate the risk model. Prospective research with a larger sample size is required to verify the clinical performance of the risk model. Besides, more functional experiments are needed to explore the potential biological mechanisms of IRFs in ccRCC.

## Data availability statement

The original contributions presented in the study are included in the article/**Supplementary Material**. Further inquiries can be directed to the corresponding author.

## Ethics statement

This study was approved by the Ethical Committee of Shandong Provincial Hospital Affiliated to Shandong First Medical University (SWYX: NO.2021-277). The patients/participants provided their written informed consent to participate in this study.

## Author contributions

HP and WL have contributed equally to this work. HP and WL conceived and designed the study, performed the experiments, performed statistical analyses and wrote the manuscript. HP and MZ interpreted data and prepared the figures. CL edited and revised the manuscript. All authors contributed to the article and approved the submitted version.

## Supplementary material

Supplementary Figure 1 The expression levels of the IRF family members between paired ccRCC samples and normal samples in the TCGA-KIRC dataset.

Supplementary Figure 2 The protein expression levels of the IRF family members between ccRCC samples and normal samples based on CPTAC using UALCAN database.

Supplementary Figure 3 Prognostic value of the IRF family members in ccRCC in the TCGA-KIRC dataset.

Supplementary Figure 4 The correlation between the IRF family members and clinical stage/histological grade.

Supplementary Figure 5 Prognostic and diagnostic values of the IRF family members in ccRCC. **(A, B)** Univariate and multivariate Cox regression analyses showed that IRF9 was an independent prognostic indicator in the TCGA-KIRC dataset. **(C)** ROC curve of IRF9 in evaluating diagnostic value for ccRCC. **(D)** ROC curve of IRF9 in predicting the 1-, 3- and 5-year OS.

Supplementary Figure 6 The distribution of clinical characteristics between the two molecular subtypes.

Supplementary Figure 7 PCA and *t*-SNE showed the distribution of the two risk groups in the TCGA-KIRC **(A, C)** and E-MTAB-1980 **(B, D)** datasets. **(E)** Differences in the expression levels of IRF family members between the low- and high-risk groups. * *p*<0.05, ** *p*<0.01, *** *p*<0.001.

Supplementary Figure 8
**(A–C)** ROC curves of the nomogram in predicting the 1-, 3-, and 5-year OS. **(D)** Distribution of the immune subtypes between the low- and high-risk groups.
