# Supplementary material for: Construction of an interferon regulatory factors-related risk model for predicting prognosis, immune microenvironment and immunotherapy in clear cell renal cell carcinoma
Source: Front Oncol. 2023 Apr 27;13:1131191. doi: 10.3389/fonc.2023.1131191 (PMC10174435; doi:10.3389/fonc.2023.1131191)
Supplement: Supplementary Figure 1 — The expression levels of the IRF family members between paired ccRCC samples and normal samples in the TCGA-KIRC dataset. [file DataSheet_1.zip › Supplementary figures/Supplementary Table S1.docx]

**Table S1. Primer sequences for qRT-PCR.**

| **Gene** | **Forward Primer** | **Reverse Primer** |
| --- | --- | --- |
| **β-actin** | TGGCACCCAGCACAATGAA | CTAAGTCATAGTCCGCCTAGAAGCA |
| **IRF1** | ATGGCTGGGACATCAACAAGG | CATGGCACAGCGAAAGTTGG |
| **IRF2** | AGTGTGCCCAGCGATGAAGA | CCCATGTTGCTGAGGTACTGTTTG |
| **IRF3** | GCTCGTGATGGTCAAGGTTGT | AGTGGGTGGCTGTTGGAAATG |
| **IRF4** | CGAAGCCTTGGCGTTCTCA | TCCAGGTTGCTGGCGTCATA |
| **IRF5** | GGACTTCCGCCTCATCTACGAC | AGGCTCAGGCTTGGCAACA |
| **IRF6** | CCCCAGGCACCTATACAGC | TCCTTCCCACGGTACTGAAAC |
| **IRF7** | CCCAGCAGGTAGCATTCCC | GCAGCAGTTCCTCCGTGTAG |
| **IRF8** | GTAGCATGTATCCAGGACTGATTTG | GCACAGCGTAACCTCGTCTTC |
| **IRF9** | CCATCTTCCACCTCACCTCTT | GGACACGATTATCACGGACAAC |
