# Supplementary material for: Construction of an interferon regulatory factors-related risk model for predicting prognosis, immune microenvironment and immunotherapy in clear cell renal cell carcinoma
Source: Front Oncol. 2023 Apr 27;13:1131191. doi: 10.3389/fonc.2023.1131191 (PMC10174435; doi:10.3389/fonc.2023.1131191)
Supplement: Supplementary Figure 1 — The expression levels of the IRF family members between paired ccRCC samples and normal samples in the TCGA-KIRC dataset. [file DataSheet_1.zip › Supplementary figures/Supplementary Table S2.docx]

**Table S2. The prognostic-related DEGs were identified by univariate Cox regression analysis.**

| **Gene** | **HR** | **HR.95L** | **HR.95H** | **P value** | **Gene** | | **HR** | **HR.95L** | **HR.95H** | **P value** |
| --- | --- | --- | --- | --- | --- | --- | --- | --- | --- | --- |
| ABHD2 | 0.65891 | 0.53963 | 0.80455 | 4.23E-05 | LIN7A | 0.74232 | | 0.66157 | 0.83293 | 3.95E-07 |
| ACAP1 | 1.42361 | 1.18449 | 1.71100 | 1.67E-04 | LINC00173 | 1.26215 | | 1.07911 | 1.47624 | 3.59E-03 |
| ACAT1 | 0.61484 | 0.52137 | 0.72506 | 7.41E-09 | LINC00342 | 1.57930 | | 1.31169 | 1.90149 | 1.41E-06 |
| ACHE | 1.53723 | 1.31921 | 1.79129 | 3.59E-08 | LINC00460 | 1.44693 | | 1.29125 | 1.62136 | 2.00E-10 |
| ACSS3 | 0.68281 | 0.58254 | 0.80034 | 2.50E-06 | LINC00475 | 1.28087 | | 1.06846 | 1.53550 | 7.45E-03 |
| ADA | 1.42971 | 1.22766 | 1.66502 | 4.26E-06 | LINC00861 | 1.41029 | | 1.09953 | 1.80887 | 6.79E-03 |
| ADAM8 | 1.70545 | 1.49027 | 1.95170 | 8.64E-15 | LINC00886 | 0.62157 | | 0.46535 | 0.83024 | 1.28E-03 |
| ADAMTS10 | 1.47717 | 1.23373 | 1.76865 | 2.18E-05 | LINC00926 | 1.87724 | | 1.50603 | 2.33995 | 2.11E-08 |
| ADAMTS14 | 1.70639 | 1.49459 | 1.94819 | 2.72E-15 | LINC00942 | 1.27922 | | 1.16228 | 1.40792 | 4.79E-07 |
| ADCY1 | 0.62681 | 0.48508 | 0.80994 | 3.55E-04 | LMO1 | 1.21598 | | 1.06453 | 1.38897 | 3.96E-03 |
| ADGRB1 | 1.28312 | 1.14756 | 1.43470 | 1.21E-05 | LNX1 | 0.64579 | | 0.51402 | 0.81133 | 1.73E-04 |
| AGBL2 | 1.67766 | 1.36507 | 2.06184 | 8.74E-07 | LRFN1 | 2.41584 | | 1.90009 | 3.07158 | 6.07E-13 |
| AGER | 1.56411 | 1.30699 | 1.87181 | 1.05E-06 | LTA | 1.46489 | | 1.12271 | 1.91137 | 4.91E-03 |
| AIM2 | 1.46062 | 1.27845 | 1.66876 | 2.49E-08 | LTB4R | 1.83964 | | 1.49287 | 2.26697 | 1.07E-08 |
| ANGPT1 | 0.76324 | 0.62988 | 0.92482 | 5.82E-03 | LTB4R2 | 1.95323 | | 1.39558 | 2.73372 | 9.49E-05 |
| ANGPTL8 | 1.18479 | 1.10027 | 1.27581 | 7.11E-06 | LY6G5B | 1.41135 | | 1.14226 | 1.74383 | 1.41E-03 |
| ANKRD13B | 1.83266 | 1.43190 | 2.34559 | 1.50E-06 | LY6H | 1.17565 | | 1.04347 | 1.32457 | 7.83E-03 |
| ANKS1A | 0.67211 | 0.51152 | 0.88310 | 4.34E-03 | MAGI3 | 0.56112 | | 0.42319 | 0.74401 | 5.96E-05 |
| ANO4 | 0.80152 | 0.68578 | 0.93680 | 5.43E-03 | MAMDC4 | 1.39061 | | 1.16031 | 1.66660 | 3.57E-04 |
| APBA2 | 1.45182 | 1.23452 | 1.70736 | 6.58E-06 | MAP4K1 | 1.31263 | | 1.09680 | 1.57093 | 3.00E-03 |
| APCDD1L | 1.28019 | 1.17546 | 1.39425 | 1.41E-08 | MARVELD2 | 0.67986 | | 0.56504 | 0.81801 | 4.35E-05 |
| APLP1 | 1.30416 | 1.14756 | 1.48212 | 4.72E-05 | MDFI | 1.28195 | | 1.10025 | 1.49367 | 1.45E-03 |
| APOBEC3D | 1.62630 | 1.30288 | 2.02999 | 1.72E-05 | MEG3 | 1.67498 | | 1.31283 | 2.13703 | 3.33E-05 |
| APOBEC3G | 1.35224 | 1.11856 | 1.63474 | 1.82E-03 | MEI1 | 1.37289 | | 1.13400 | 1.66210 | 1.16E-03 |
| APOBEC3H | 1.45730 | 1.19825 | 1.77234 | 1.62E-04 | MELTF | 1.51151 | | 1.35671 | 1.68398 | 6.70E-14 |
| APOL1 | 1.21521 | 1.09147 | 1.35298 | 3.75E-04 | MFSD2A | 1.32947 | | 1.18931 | 1.48616 | 5.44E-07 |
| AQP9 | 1.20261 | 1.09966 | 1.31519 | 5.33E-05 | MIAT | 1.38412 | | 1.22797 | 1.56013 | 1.02E-07 |
| ARHGAP33 | 1.49529 | 1.22818 | 1.82049 | 6.15E-05 | MICALL2 | 1.94040 | | 1.58425 | 2.37661 | 1.48E-10 |
| ARHGAP6 | 0.49322 | 0.35704 | 0.68133 | 1.81E-05 | MIR155HG | 1.37744 | | 1.19010 | 1.59427 | 1.76E-05 |
| ARHGAP9 | 1.42298 | 1.18101 | 1.71452 | 2.08E-04 | MIR503HG | 1.29781 | | 1.12417 | 1.49827 | 3.75E-04 |
| ASTN2 | 0.41890 | 0.30095 | 0.58308 | 2.51E-07 | MMP1 | 1.14398 | | 1.04386 | 1.25370 | 4.00E-03 |
| ATAD3B | 2.15919 | 1.68651 | 2.76435 | 1.02E-09 | MOCOS | 1.50231 | | 1.33360 | 1.69237 | 2.14E-11 |
| ATG16L2 | 1.40664 | 1.16639 | 1.69636 | 3.56E-04 | MPP7 | 0.45958 | | 0.32638 | 0.64715 | 8.50E-06 |
| ATP1A1 | 0.55090 | 0.41861 | 0.72498 | 2.09E-05 | MPPED2 | 0.47204 | | 0.30715 | 0.72545 | 6.17E-04 |
| ATP6V1A | 0.62000 | 0.48827 | 0.78726 | 8.75E-05 | MSC | 1.39387 | | 1.22975 | 1.57990 | 2.04E-07 |
| ATP8B3 | 1.27912 | 1.13461 | 1.44204 | 5.71E-05 | MSH5 | 1.67707 | | 1.27885 | 2.19928 | 1.85E-04 |
| AURKB | 1.81163 | 1.56505 | 2.09706 | 1.72E-15 | MSTO2P | 1.92268 | | 1.51330 | 2.44282 | 8.73E-08 |
| BACE2 | 1.25324 | 1.10555 | 1.42065 | 4.18E-04 | MT1DP | 1.25298 | | 1.09517 | 1.43353 | 1.03E-03 |
| BASP1 | 1.42298 | 1.25750 | 1.61023 | 2.24E-08 | MT1X | 1.20667 | | 1.10644 | 1.31599 | 2.18E-05 |
| BATF | 1.34207 | 1.16047 | 1.55210 | 7.30E-05 | MT2A | 1.32016 | | 1.18319 | 1.47298 | 6.70E-07 |
| BATF3 | 1.43305 | 1.14301 | 1.79668 | 1.82E-03 | MTMR9LP | 1.30758 | | 1.10886 | 1.54191 | 1.43E-03 |
| BCL3 | 1.93013 | 1.58012 | 2.35766 | 1.18E-10 | MTURN | 0.59409 | | 0.48149 | 0.73302 | 1.19E-06 |
| BEST4 | 1.21958 | 1.07098 | 1.38879 | 2.75E-03 | MUC20 | 0.83732 | | 0.74324 | 0.94331 | 3.50E-03 |
| BGLAP | 1.99825 | 1.49286 | 2.67473 | 3.26E-06 | MXD3 | 2.54143 | | 1.98311 | 3.25694 | 1.71E-13 |
| BSPRY | 0.68468 | 0.59619 | 0.78631 | 8.12E-08 | MZB1 | 1.19322 | | 1.09715 | 1.29770 | 3.71E-05 |
| BTBD19 | 1.50714 | 1.22889 | 1.84840 | 8.18E-05 | NBL1 | 1.23287 | | 1.08423 | 1.40188 | 1.40E-03 |
| C16orf74 | 1.17705 | 1.05976 | 1.30733 | 2.34E-03 | NCALD | 0.61409 | | 0.45317 | 0.83216 | 1.66E-03 |
| C1orf53 | 1.32936 | 1.15123 | 1.53506 | 1.05E-04 | NCR3LG1 | 0.56355 | | 0.45614 | 0.69626 | 1.06E-07 |
| C1QL1 | 1.12530 | 1.04748 | 1.20892 | 1.24E-03 | NEDD4L | 0.62816 | | 0.49722 | 0.79359 | 9.69E-05 |
| C1QTNF6 | 1.91877 | 1.56220 | 2.35672 | 5.20E-10 | NFKBIZ | 1.49702 | | 1.30854 | 1.71265 | 4.19E-09 |
| C1R | 1.37504 | 1.22969 | 1.53758 | 2.31E-08 | NNMT | 1.21067 | | 1.06356 | 1.37812 | 3.82E-03 |
| C1S | 1.33043 | 1.19558 | 1.48049 | 1.64E-07 | NNT | 0.58356 | | 0.47014 | 0.72435 | 1.04E-06 |
| C2CD4B | 1.21376 | 1.06169 | 1.38760 | 4.56E-03 | NOD2 | 1.67975 | | 1.36912 | 2.06086 | 6.65E-07 |
| C2orf88 | 0.72724 | 0.58221 | 0.90840 | 5.01E-03 | NPEPL1 | 1.80094 | | 1.54098 | 2.10476 | 1.40E-13 |
| CA2 | 0.71486 | 0.61505 | 0.83085 | 1.21E-05 | NPIPB5 | 1.93939 | | 1.47552 | 2.54909 | 2.04E-06 |
| CACNA1D | 0.65046 | 0.48743 | 0.86803 | 3.49E-03 | NPNT | 0.68290 | | 0.59319 | 0.78617 | 1.11E-07 |
| CACNA2D4 | 1.26190 | 1.09671 | 1.45197 | 1.16E-03 | NR3C2 | 0.51569 | | 0.41607 | 0.63917 | 1.48E-09 |
| CARD11 | 1.26603 | 1.09480 | 1.46404 | 1.47E-03 | NRXN3 | 0.68375 | | 0.53006 | 0.88201 | 3.43E-03 |
| CARD9 | 1.85393 | 1.42418 | 2.41337 | 4.48E-06 | NUMBL | 2.20444 | | 1.84114 | 2.63941 | 7.75E-18 |
| CARMIL2 | 1.85119 | 1.40276 | 2.44296 | 1.35E-05 | OCLN | 0.63793 | | 0.51685 | 0.78737 | 2.84E-05 |
| CASR | 0.65496 | 0.50505 | 0.84936 | 1.42E-03 | ODF3B | 1.40629 | | 1.17608 | 1.68157 | 1.85E-04 |
| CCDC74B | 1.54562 | 1.25286 | 1.90679 | 4.82E-05 | OGDHL | 0.79503 | | 0.71125 | 0.88868 | 5.41E-05 |
| CCDC88B | 1.59778 | 1.29478 | 1.97168 | 1.25E-05 | OLFM2 | 1.26721 | | 1.07629 | 1.49199 | 4.48E-03 |
| CCL19 | 1.12022 | 1.03246 | 1.21544 | 6.38E-03 | OVOL1 | 0.69487 | | 0.57996 | 0.83254 | 7.91E-05 |
| CCL5 | 1.16495 | 1.04226 | 1.30209 | 7.17E-03 | P4HA3 | 1.34137 | | 1.18999 | 1.51202 | 1.53E-06 |
| CCNL2 | 1.44938 | 1.24669 | 1.68503 | 1.37E-06 | PABPC1L | 1.63393 | | 1.42497 | 1.87354 | 2.03E-12 |
| CD68 | 1.51386 | 1.23194 | 1.86031 | 8.02E-05 | PADI3 | 1.17915 | | 1.05562 | 1.31714 | 3.52E-03 |
| CD7 | 1.39150 | 1.21333 | 1.59585 | 2.29E-06 | PAEP | 1.15356 | | 1.07902 | 1.23325 | 2.77E-05 |
| CD72 | 1.41198 | 1.19618 | 1.66672 | 4.57E-05 | PAGE2B | 1.24203 | | 1.08299 | 1.42443 | 1.93E-03 |
| CD9 | 0.72342 | 0.60569 | 0.86404 | 3.54E-04 | PAQR6 | 1.58032 | | 1.31696 | 1.89633 | 8.65E-07 |
| CDCA3 | 2.31218 | 1.92279 | 2.78042 | 5.18E-19 | PARP15 | 1.46847 | | 1.17881 | 1.82931 | 6.09E-04 |
| CDH1 | 0.78984 | 0.69616 | 0.89613 | 2.50E-04 | PARVG | 1.51547 | | 1.21199 | 1.89495 | 2.66E-04 |
| CDH16 | 0.76912 | 0.69252 | 0.85419 | 9.38E-07 | PBX4 | 1.69354 | | 1.35103 | 2.12288 | 4.88E-06 |
| CDKN2A | 1.46738 | 1.22302 | 1.76058 | 3.69E-05 | PCED1B-AS1 | 1.48071 | | 1.19255 | 1.83851 | 3.78E-04 |
| CDS1 | 0.61333 | 0.51333 | 0.73280 | 7.31E-08 | PDGFRL | 1.31628 | | 1.18916 | 1.45699 | 1.14E-07 |
| CEACAM4 | 1.70660 | 1.39831 | 2.08285 | 1.46E-07 | PGGHG | 1.20549 | | 1.09479 | 1.32738 | 1.43E-04 |
| CES3 | 0.87046 | 0.78965 | 0.95955 | 5.26E-03 | PI3 | 1.22707 | | 1.13615 | 1.32526 | 1.89E-07 |
| CGN | 0.65212 | 0.55773 | 0.76249 | 8.37E-08 | PIK3R6 | 1.46630 | | 1.24183 | 1.73134 | 6.34E-06 |
| CIDEC | 1.22003 | 1.06799 | 1.39371 | 3.40E-03 | PIM2 | 1.33637 | | 1.16165 | 1.53737 | 4.99E-05 |
| CIPC | 0.60012 | 0.44671 | 0.80622 | 6.99E-04 | PIP5K1B | 0.64973 | | 0.48748 | 0.86598 | 3.27E-03 |
| CLDND2 | 1.74653 | 1.38531 | 2.20195 | 2.40E-06 | PKMYT1 | 2.14671 | | 1.67637 | 2.74903 | 1.41E-09 |
| CLIC5 | 0.61372 | 0.49683 | 0.75813 | 5.94E-06 | PLA2G2A | 1.33320 | | 1.18983 | 1.49383 | 7.25E-07 |
| CMTM4 | 0.69776 | 0.56590 | 0.86035 | 7.59E-04 | PLAC8 | 1.48422 | | 1.19606 | 1.84182 | 3.36E-04 |
| CNGA1 | 0.44769 | 0.30270 | 0.66215 | 5.71E-05 | PLAC8L1 | 1.66542 | | 1.30742 | 2.12144 | 3.62E-05 |
| COBLL1 | 0.67945 | 0.51968 | 0.88834 | 4.72E-03 | PLCB2 | 1.44671 | | 1.18915 | 1.76007 | 2.23E-04 |
| COL22A1 | 1.53131 | 1.34788 | 1.73969 | 5.91E-11 | PLCG2 | 0.67484 | | 0.50527 | 0.90134 | 7.73E-03 |
| COL4A4 | 0.57411 | 0.46741 | 0.70518 | 1.23E-07 | PLCL2 | 0.49312 | | 0.39315 | 0.61851 | 9.57E-10 |
| COLCA1 | 0.70848 | 0.57283 | 0.87626 | 1.48E-03 | PLEKHG4 | 1.42571 | | 1.20734 | 1.68358 | 2.90E-05 |
| CPA4 | 1.28797 | 1.15026 | 1.44216 | 1.15E-05 | PLIN1 | 1.44349 | | 1.18654 | 1.75608 | 2.42E-04 |
| CPNE7 | 1.40916 | 1.26661 | 1.56774 | 2.91E-10 | PLLP | 0.62424 | | 0.49299 | 0.79043 | 9.13E-05 |
| CRHBP | 0.61915 | 0.47865 | 0.80091 | 2.62E-04 | PLXNB3 | 1.53829 | | 1.35459 | 1.74690 | 3.19E-11 |
| CRP | 1.20004 | 1.09105 | 1.31992 | 1.74E-04 | POU2AF1 | 1.19289 | | 1.06938 | 1.33067 | 1.56E-03 |
| CRYBG3 | 0.66649 | 0.49735 | 0.89316 | 6.60E-03 | POU2F2 | 1.57957 | | 1.28151 | 1.94697 | 1.83E-05 |
| CSF3R | 1.43486 | 1.17574 | 1.75110 | 3.81E-04 | PPARGC1A | 0.67369 | | 0.57100 | 0.79485 | 2.85E-06 |
| CTHRC1 | 1.22282 | 1.10794 | 1.34961 | 6.43E-05 | PPM1L | 0.72261 | | 0.56618 | 0.92227 | 9.05E-03 |
| CTLA4 | 1.47530 | 1.22741 | 1.77326 | 3.43E-05 | PPP1R1A | 1.20027 | | 1.12196 | 1.28405 | 1.14E-07 |
| CXCL13 | 1.19934 | 1.10223 | 1.30501 | 2.45E-05 | PRAME | 1.21156 | | 1.11492 | 1.31658 | 6.04E-06 |
| CXCL2 | 1.31003 | 1.18310 | 1.45058 | 2.06E-07 | PRDM8 | 1.56831 | | 1.26827 | 1.93935 | 3.27E-05 |
| CXCL3 | 1.41079 | 1.22663 | 1.62260 | 1.42E-06 | PRR15L | 0.83129 | | 0.74514 | 0.92741 | 9.33E-04 |
| CXCL5 | 1.21095 | 1.11745 | 1.31227 | 3.03E-06 | PRR22 | 1.44201 | | 1.12720 | 1.84474 | 3.58E-03 |
| CYP21A2 | 1.75214 | 1.43813 | 2.13471 | 2.61E-08 | PRR7 | 1.31646 | | 1.07894 | 1.60627 | 6.76E-03 |
| CYP39A1 | 0.75245 | 0.60635 | 0.93376 | 9.82E-03 | PRRT2 | 1.45850 | | 1.19638 | 1.77805 | 1.89E-04 |
| DBH-AS1 | 1.74449 | 1.48311 | 2.05194 | 1.83E-11 | PRSS53 | 1.95376 | | 1.56373 | 2.44108 | 3.75E-09 |
| DDIT4L | 0.83547 | 0.74733 | 0.93400 | 1.58E-03 | PSTPIP1 | 1.39123 | | 1.15786 | 1.67164 | 4.24E-04 |
| DEF6 | 1.47588 | 1.22215 | 1.78229 | 5.25E-05 | PTCH2 | 1.70612 | | 1.26518 | 2.30073 | 4.62E-04 |
| DERL3 | 1.35371 | 1.17763 | 1.55611 | 2.05E-05 | PTGER3 | 0.86428 | | 0.78705 | 0.94908 | 2.26E-03 |
| DGCR10 | 1.25812 | 1.07060 | 1.47849 | 5.30E-03 | PTK6 | 1.28620 | | 1.14021 | 1.45088 | 4.23E-05 |
| DGKI | 0.42196 | 0.27572 | 0.64579 | 7.07E-05 | PTPN7 | 1.30453 | | 1.10083 | 1.54593 | 2.15E-03 |
| DLK2 | 1.32457 | 1.14732 | 1.52921 | 1.26E-04 | PTPRH | 1.24847 | | 1.15276 | 1.35212 | 4.94E-08 |
| DOK3 | 1.86179 | 1.47150 | 2.35560 | 2.24E-07 | PTPRN | 1.18825 | | 1.05739 | 1.33531 | 3.76E-03 |
| DSG2 | 0.72704 | 0.62190 | 0.84996 | 6.34E-05 | PWAR5 | 0.42121 | | 0.26659 | 0.66552 | 2.12E-04 |
| DUSP5P1 | 1.65344 | 1.37861 | 1.98304 | 5.90E-08 | PYCARD | 1.49652 | | 1.26733 | 1.76717 | 2.00E-06 |
| EBI3 | 1.38671 | 1.15765 | 1.66110 | 3.86E-04 | PYCR1 | 1.49387 | | 1.33082 | 1.67689 | 9.98E-12 |
| EFHD1 | 0.78228 | 0.68263 | 0.89647 | 4.12E-04 | RAB24 | 1.40140 | | 1.12186 | 1.75058 | 2.95E-03 |
| EGFL8 | 1.58087 | 1.23109 | 2.03003 | 3.31E-04 | RAB27B | 1.43011 | | 1.17857 | 1.73535 | 2.89E-04 |
| EIF4A1 | 1.53985 | 1.25965 | 1.88238 | 2.53E-05 | RAP1GAP | 0.80914 | | 0.69704 | 0.93927 | 5.38E-03 |
| EIF4EBP1 | 1.50066 | 1.29750 | 1.73564 | 4.53E-08 | RARRES1 | 1.19932 | | 1.09904 | 1.30875 | 4.51E-05 |
| EME2 | 1.56690 | 1.29622 | 1.89410 | 3.46E-06 | RASAL3 | 1.35383 | | 1.08870 | 1.68351 | 6.44E-03 |
| ENAM | 0.51516 | 0.39727 | 0.66805 | 5.67E-07 | RASGEF1A | 1.31661 | | 1.09716 | 1.57995 | 3.11E-03 |
| ENPP5 | 0.61198 | 0.52493 | 0.71346 | 3.55E-10 | RETN | 1.24826 | | 1.09462 | 1.42346 | 9.36E-04 |
| EPCAM | 0.84085 | 0.75229 | 0.93983 | 2.27E-03 | RHEBL1 | 1.97342 | | 1.52386 | 2.55562 | 2.56E-07 |
| ERMP1 | 0.53316 | 0.41050 | 0.69247 | 2.42E-06 | RIN1 | 1.78549 | | 1.46321 | 2.17876 | 1.15E-08 |
| ESPL1 | 1.68657 | 1.28212 | 2.21860 | 1.87E-04 | RNASE2 | 1.54803 | | 1.33073 | 1.80081 | 1.49E-08 |
| ESRRG | 0.60271 | 0.47286 | 0.76823 | 4.32E-05 | RNASET2 | 1.26367 | | 1.11703 | 1.42957 | 2.00E-04 |
| EXPH5 | 0.67165 | 0.51309 | 0.87920 | 3.77E-03 | RNF152 | 0.67289 | | 0.58339 | 0.77612 | 5.32E-08 |
| FADS3 | 1.98224 | 1.54568 | 2.54209 | 7.01E-08 | RNF180 | 0.74066 | | 0.62145 | 0.88274 | 8.00E-04 |
| FAM160A1 | 0.34456 | 0.24246 | 0.48966 | 2.81E-09 | RNF43 | 0.70340 | | 0.55975 | 0.88392 | 2.54E-03 |
| FAM193B | 1.48897 | 1.26495 | 1.75266 | 1.71E-06 | RPL23AP32 | 1.37461 | | 1.18878 | 1.58950 | 1.76E-05 |
| FCGR1B | 1.95064 | 1.51471 | 2.51202 | 2.25E-07 | SAA1 | 1.13764 | | 1.09193 | 1.18527 | 7.12E-10 |
| FCGR2C | 1.38118 | 1.15952 | 1.64521 | 2.97E-04 | SAPCD1 | 1.98922 | | 1.56807 | 2.52348 | 1.46E-08 |
| FCHO1 | 1.50052 | 1.24737 | 1.80505 | 1.67E-05 | SC5D | 0.56194 | | 0.42549 | 0.74216 | 4.89E-05 |
| FDCSP | 1.15664 | 1.07614 | 1.24316 | 7.70E-05 | SCD5 | 0.72718 | | 0.63331 | 0.83495 | 6.24E-06 |
| FER1L4 | 1.39429 | 1.20857 | 1.60855 | 5.18E-06 | SCNN1D | 1.61159 | | 1.30720 | 1.98687 | 7.89E-06 |
| FGF1 | 0.70200 | 0.54569 | 0.90309 | 5.90E-03 | SEC31B | 1.88354 | | 1.46465 | 2.42222 | 8.07E-07 |
| FKBP10 | 1.45317 | 1.27053 | 1.66206 | 4.93E-08 | SERPINE1 | 1.14585 | | 1.04613 | 1.25508 | 3.38E-03 |
| FKBP11 | 1.87333 | 1.58404 | 2.21545 | 2.22E-13 | SH2D2A | 1.38179 | | 1.18145 | 1.61610 | 5.20E-05 |
| FLT3LG | 1.98546 | 1.44966 | 2.71930 | 1.92E-05 | SH2D4A | 0.63497 | | 0.49002 | 0.82280 | 5.92E-04 |
| FMNL1 | 1.60322 | 1.28857 | 1.99469 | 2.29E-05 | SHROOM3 | 0.63074 | | 0.51240 | 0.77641 | 1.38E-05 |
| FOSL1 | 1.31925 | 1.17013 | 1.48738 | 5.98E-06 | SLC11A1 | 1.82044 | | 1.50079 | 2.20817 | 1.19E-09 |
| FOXP3 | 1.52061 | 1.23396 | 1.87386 | 8.41E-05 | SLC17A9 | 1.52079 | | 1.33750 | 1.72918 | 1.57E-10 |
| FUT1 | 0.56917 | 0.43453 | 0.74554 | 4.27E-05 | SLC18A3 | 1.25411 | | 1.14116 | 1.37825 | 2.58E-06 |
| GABRE | 1.23262 | 1.07972 | 1.40718 | 1.97E-03 | SLC25A4 | 0.47687 | | 0.37402 | 0.60801 | 2.31E-09 |
| GATA2 | 0.73706 | 0.59595 | 0.91159 | 4.90E-03 | SLC38A5 | 1.26489 | | 1.17035 | 1.36706 | 3.05E-09 |
| GATA2-AS1 | 0.65512 | 0.48912 | 0.87747 | 4.56E-03 | SLC4A3 | 1.29415 | | 1.15200 | 1.45384 | 1.40E-05 |
| GCNT1 | 1.42909 | 1.16543 | 1.75241 | 6.01E-04 | SLC6A3 | 0.89326 | | 0.83627 | 0.95412 | 7.90E-04 |
| GFI1 | 1.33059 | 1.08692 | 1.62890 | 5.65E-03 | SNHG12 | 1.39105 | | 1.19234 | 1.62287 | 2.71E-05 |
| GFPT2 | 1.43157 | 1.29265 | 1.58541 | 5.62E-12 | SNHG25 | 1.45009 | | 1.21247 | 1.73427 | 4.70E-05 |
| GJB2 | 1.25806 | 1.11372 | 1.42111 | 2.22E-04 | SNHG3 | 1.89752 | | 1.57397 | 2.28758 | 1.87E-11 |
| GOLGA8A | 1.29047 | 1.13559 | 1.46647 | 9.26E-05 | SORL1 | 0.62251 | | 0.51883 | 0.74690 | 3.41E-07 |
| GPAT3 | 0.69741 | 0.59047 | 0.82371 | 2.20E-05 | SOWAHD | 1.49971 | | 1.15489 | 1.94748 | 2.36E-03 |
| GPD1L | 0.57965 | 0.43539 | 0.77170 | 1.88E-04 | SOX6 | 0.59114 | | 0.47587 | 0.73433 | 2.03E-06 |
| GPR84 | 1.83602 | 1.51432 | 2.22605 | 6.32E-10 | SP140 | 1.53048 | | 1.22512 | 1.91196 | 1.78E-04 |
| GPRIN1 | 1.60777 | 1.27764 | 2.02320 | 5.14E-05 | SPHK1 | 1.69415 | | 1.42311 | 2.01682 | 3.09E-09 |
| GPS2 | 1.71242 | 1.35061 | 2.17116 | 8.92E-06 | SPINK13 | 1.12439 | | 1.04577 | 1.20893 | 1.53E-03 |
| GSDMB | 1.97806 | 1.60812 | 2.43309 | 1.07E-10 | SPOCK1 | 1.14333 | | 1.05442 | 1.23974 | 1.18E-03 |
| HACD3 | 0.66867 | 0.51044 | 0.87595 | 3.49E-03 | SRPX2 | 1.26783 | | 1.16573 | 1.37887 | 3.03E-08 |
| HAMP | 1.52884 | 1.32053 | 1.77002 | 1.35E-08 | STEAP3 | 1.33301 | | 1.21452 | 1.46306 | 1.43E-09 |
| HAPLN3 | 1.60851 | 1.36185 | 1.89985 | 2.19E-08 | STX1B | 1.66546 | | 1.22660 | 2.26132 | 1.08E-03 |
| HCST | 1.42051 | 1.22333 | 1.64946 | 4.15E-06 | SYCE1L | 1.62064 | | 1.37551 | 1.90946 | 7.91E-09 |
| HDAC10 | 2.13041 | 1.64652 | 2.75650 | 8.75E-09 | TBC1D1 | 0.47044 | | 0.32205 | 0.68720 | 9.61E-05 |
| HJURP | 1.93949 | 1.65991 | 2.26615 | 7.37E-17 | TBC1D10C | 1.36138 | | 1.13928 | 1.62678 | 6.86E-04 |
| HMGCS2 | 0.83744 | 0.77379 | 0.90632 | 1.09E-05 | TBC1D14 | 0.63187 | | 0.50864 | 0.78496 | 3.36E-05 |
| HOXB9 | 1.18917 | 1.07587 | 1.31441 | 6.95E-04 | TBC1D4 | 0.61593 | | 0.49020 | 0.77391 | 3.18E-05 |
| HPGD | 0.74898 | 0.63353 | 0.88546 | 7.14E-04 | TFPI2 | 1.13718 | | 1.05615 | 1.22442 | 6.53E-04 |
| HSD11B2 | 0.80202 | 0.71794 | 0.89595 | 9.44E-05 | TGFBI | 1.12779 | | 1.05283 | 1.20808 | 6.10E-04 |
| HSD17B3 | 1.50992 | 1.26140 | 1.80741 | 7.09E-06 | THRB | 0.44519 | | 0.32853 | 0.60327 | 1.79E-07 |
| HSD3B7 | 1.25613 | 1.07218 | 1.47165 | 4.76E-03 | THSD7A | 0.64736 | | 0.52052 | 0.80511 | 9.30E-05 |
| HSH2D | 1.60746 | 1.33457 | 1.93614 | 5.72E-07 | TIGIT | 1.25153 | | 1.06852 | 1.46589 | 5.41E-03 |
| IFFO1 | 2.07232 | 1.60501 | 2.67570 | 2.29E-08 | TMC8 | 1.39010 | | 1.14716 | 1.68449 | 7.77E-04 |
| IFI30 | 1.81054 | 1.48499 | 2.20747 | 4.37E-09 | TMEM44 | 1.88749 | | 1.53810 | 2.31623 | 1.18E-09 |
| IFITM4P | 1.73844 | 1.34468 | 2.24751 | 2.44E-05 | TMEM8B | 0.63451 | | 0.48251 | 0.83439 | 1.13E-03 |
| IFNG | 1.31391 | 1.13165 | 1.52552 | 3.39E-04 | TNFAIP2 | 1.47943 | | 1.23196 | 1.77660 | 2.75E-05 |
| IGFLR1 | 1.97757 | 1.60599 | 2.43513 | 1.35E-10 | TNFRSF17 | 1.24034 | | 1.08180 | 1.42212 | 2.02E-03 |
| IL1R2 | 1.15783 | 1.07198 | 1.25056 | 1.93E-04 | TNFRSF18 | 1.57777 | | 1.31936 | 1.88678 | 5.83E-07 |
| IL20RB | 1.20426 | 1.13148 | 1.28172 | 5.10E-09 | TNFRSF25 | 1.48182 | | 1.22327 | 1.79503 | 5.82E-05 |
| IL21R | 1.40945 | 1.13821 | 1.74533 | 1.65E-03 | TNFSF14 | 1.48982 | | 1.30744 | 1.69765 | 2.19E-09 |
| IL6 | 1.18585 | 1.10796 | 1.26922 | 8.77E-07 | TNNT1 | 1.23716 | | 1.14644 | 1.33507 | 4.33E-08 |
| ILDR1 | 0.65520 | 0.52106 | 0.82389 | 2.98E-04 | TOX3 | 0.61233 | | 0.50408 | 0.74382 | 7.74E-07 |
| INHBE | 1.30967 | 1.17247 | 1.46294 | 1.77E-06 | TPSG1 | 1.21635 | | 1.09492 | 1.35124 | 2.62E-04 |
| INPP4B | 0.62834 | 0.49116 | 0.80383 | 2.18E-04 | TRAF1 | 1.39977 | | 1.14128 | 1.71680 | 1.24E-03 |
| IRF4 | 1.26383 | 1.08315 | 1.47465 | 2.93E-03 | TREM1 | 1.34790 | | 1.19726 | 1.51750 | 7.92E-07 |
| IRF6 | 0.66898 | 0.58595 | 0.76377 | 2.75E-09 | TRIB3 | 1.36400 | | 1.21348 | 1.53320 | 1.96E-07 |
| IRF7 | 1.67522 | 1.37772 | 2.03695 | 2.31E-07 | TRIM2 | 0.57135 | | 0.46808 | 0.69740 | 3.73E-08 |
| IRF9 | 1.69021 | 1.36302 | 2.09593 | 1.74E-06 | TRIM46 | 1.71137 | | 1.41810 | 2.06530 | 2.12E-08 |
| ISG20 | 1.64136 | 1.30617 | 2.06257 | 2.12E-05 | TROAP | 2.04735 | | 1.72156 | 2.43478 | 5.35E-16 |
| ITPKA | 1.38077 | 1.26460 | 1.50761 | 6.24E-13 | TSNAXIP1 | 1.50120 | | 1.13964 | 1.97745 | 3.86E-03 |
| IZUMO4 | 1.87794 | 1.49447 | 2.35981 | 6.39E-08 | TSPAN32 | 1.47796 | | 1.15273 | 1.89494 | 2.06E-03 |
| JAK3 | 1.69139 | 1.40971 | 2.02936 | 1.56E-08 | TSPAN7 | 0.69134 | | 0.61331 | 0.77930 | 1.53E-09 |
| JSRP1 | 1.35577 | 1.19469 | 1.53857 | 2.40E-06 | TUBB2B | 1.25204 | | 1.09460 | 1.43213 | 1.04E-03 |
| KAT2A | 1.76776 | 1.46374 | 2.13493 | 3.28E-09 | TYMP | 1.47836 | | 1.21401 | 1.80027 | 1.01E-04 |
| KCNK17 | 1.26576 | 1.12442 | 1.42487 | 9.57E-05 | UBE2QL1 | 0.74076 | | 0.61634 | 0.89029 | 1.38E-03 |
| KDF1 | 0.57662 | 0.46884 | 0.70916 | 1.83E-07 | UBL3 | 0.69786 | | 0.54283 | 0.89717 | 5.01E-03 |
| KIF13B | 0.52500 | 0.39629 | 0.69552 | 7.11E-06 | UBXN11 | 1.38136 | | 1.12130 | 1.70174 | 2.40E-03 |
| KIF18B | 2.43717 | 1.95745 | 3.03445 | 1.64E-15 | UCN | 2.13079 | | 1.71516 | 2.64715 | 8.31E-12 |
| KIF21A | 0.74944 | 0.61154 | 0.91845 | 5.44E-03 | UGT8 | 0.71903 | | 0.60203 | 0.85877 | 2.72E-04 |
| KISS1 | 1.50647 | 1.27486 | 1.78017 | 1.50E-06 | UNC13D | 1.83499 | | 1.49481 | 2.25258 | 6.53E-09 |
| KIT | 0.78588 | 0.65730 | 0.93962 | 8.21E-03 | VENTX | 1.54193 | | 1.21464 | 1.95741 | 3.75E-04 |
| KLF5 | 0.74903 | 0.62069 | 0.90390 | 2.58E-03 | WAS | 1.37146 | | 1.13035 | 1.66400 | 1.36E-03 |
| KLHL17 | 1.86010 | 1.43182 | 2.41648 | 3.35E-06 | WDR72 | 0.63954 | | 0.56704 | 0.72130 | 3.29E-13 |
| LAG3 | 1.20057 | 1.07824 | 1.33678 | 8.56E-04 | XCL1 | 1.38758 | | 1.16769 | 1.64888 | 1.98E-04 |
| LAIR2 | 1.24049 | 1.09702 | 1.40272 | 5.89E-04 | YJEFN3 | 1.70510 | | 1.37927 | 2.10789 | 8.14E-07 |
| LARS2 | 0.58039 | 0.40664 | 0.82838 | 2.72E-03 | ZAP70 | 1.39843 | | 1.15945 | 1.68668 | 4.53E-04 |
| LBP | 1.12978 | 1.07424 | 1.18819 | 2.09E-06 | ZBED2 | 1.28563 | | 1.11635 | 1.48058 | 4.87E-04 |
| LBX2 | 2.87470 | 2.10135 | 3.93266 | 3.99E-11 | ZBP1 | 1.57324 | | 1.29429 | 1.91232 | 5.35E-06 |
| LCAT | 1.38059 | 1.15437 | 1.65113 | 4.12E-04 | ZBTB20 | 0.36564 | | 0.20922 | 0.63899 | 4.12E-04 |
| LDHD | 0.73237 | 0.62916 | 0.85251 | 5.85E-05 | ZNF114 | 1.15752 | | 1.04241 | 1.28534 | 6.20E-03 |
| LGALS12 | 1.15224 | 1.04251 | 1.27351 | 5.51E-03 | ZNF296 | 1.50363 | | 1.15896 | 1.95079 | 2.14E-03 |
| LIF | 1.30805 | 1.16717 | 1.46594 | 3.86E-06 | ZNF683 | 1.33736 | | 1.13322 | 1.57826 | 5.82E-04 |
| LILRB3 | 1.87551 | 1.48386 | 2.37052 | 1.42E-07 | ZNF692 | 1.63194 | | 1.36047 | 1.95758 | 1.32E-07 |
| LIMD2 | 1.47206 | 1.23635 | 1.75270 | 1.41E-05 | ZP3 | 1.33513 | | 1.13561 | 1.56970 | 4.65E-04 |
| LIME1 | 1.60966 | 1.25536 | 2.06395 | 1.75E-04 |  |  | |  |  |  |
