# Supplementary material for: Construction of an interferon regulatory factors-related risk model for predicting prognosis, immune microenvironment and immunotherapy in clear cell renal cell carcinoma
Source: Front Oncol. 2023 Apr 27;13:1131191. doi: 10.3389/fonc.2023.1131191 (PMC10174435; doi:10.3389/fonc.2023.1131191)
Supplement: Supplementary Figure 1 — The expression levels of the IRF family members between paired ccRCC samples and normal samples in the TCGA-KIRC dataset. [file DataSheet_1.zip › Supplementary figures/Supplementary Figure S2.pdf]

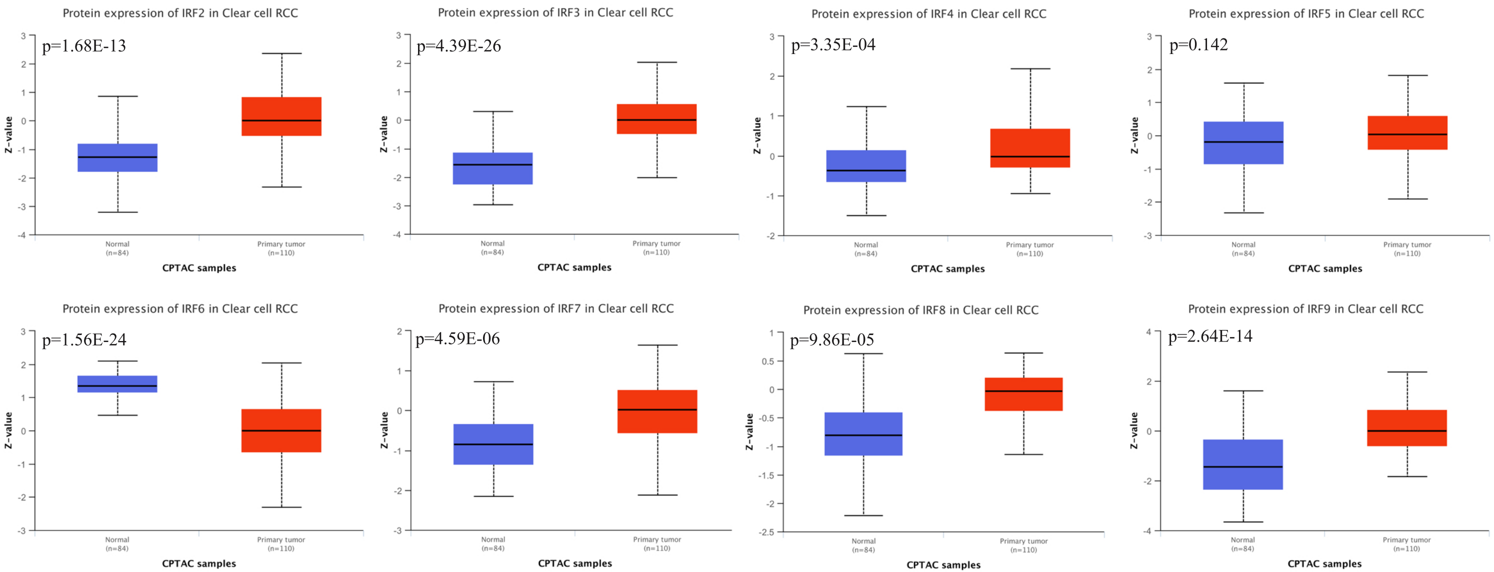

Protein expression of IRF2 in Clear cell RCC
p=1.68E-13
Protein expression of IRF3 in Clear cell RCC
p=4.39E-26
Protein expression of IRF4 in Clear cell RCC
p=3.35E-04
Protein expression of IRF5 in Clear cell RCC
p=0.142
Protein expression of IRF6 in Clear cell RCC
p=1.56E-24
Protein expression of IRF7 in Clear cell RCC
p=4.59E-06
Protein expression of IRF8 in Clear cell RCC
p=9.86E-05
Protein expression of IRF9 in Clear cell RCC
p=2.64E-14
Z-value
Normal (n=84)
Primary tumor (n=110)
CPTAC samples
