# Supplementary figures and images for: Construction of an interferon regulatory factors-related risk model for predicting prognosis, immune microenvironment and immunotherapy in clear cell renal cell carcinoma
Source: Front Oncol. 2023 Apr 27;13:1131191. doi: 10.3389/fonc.2023.1131191 (PMC10174435; doi:10.3389/fonc.2023.1131191)

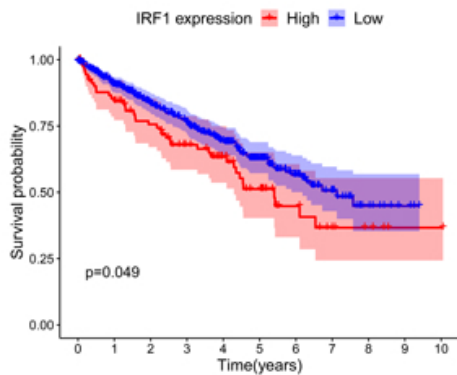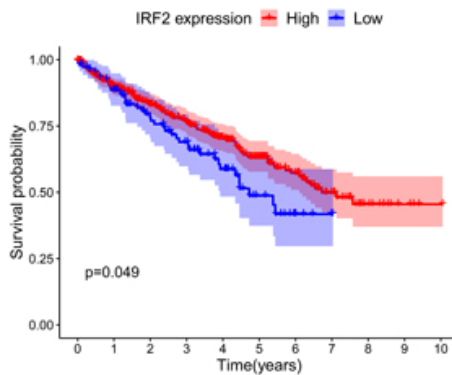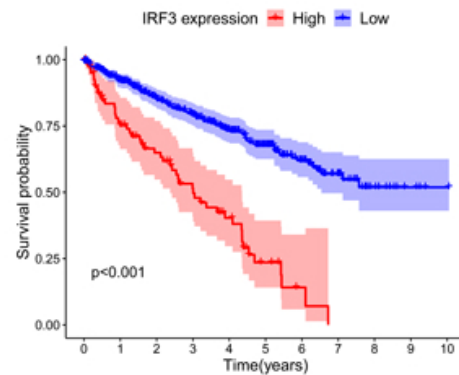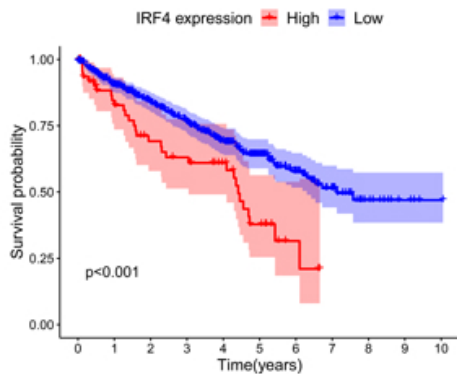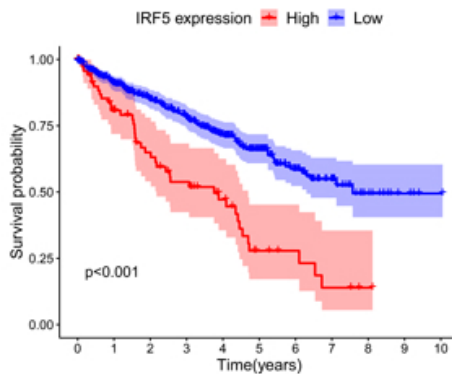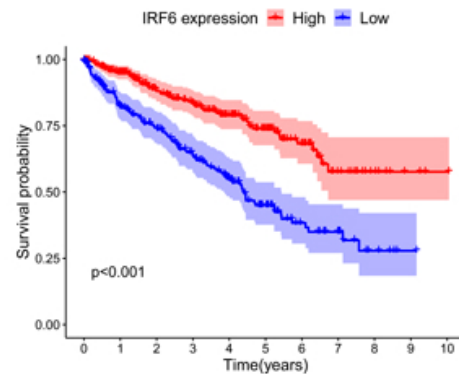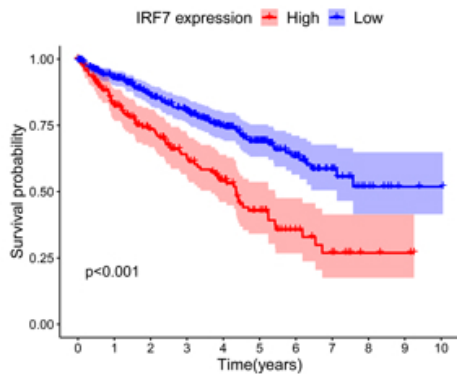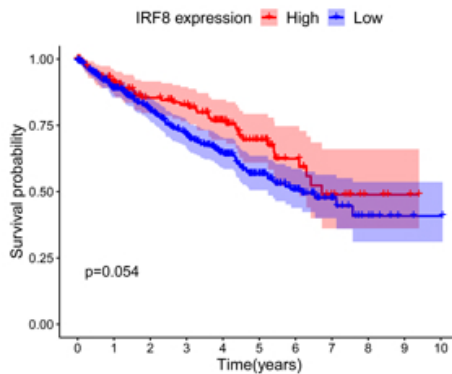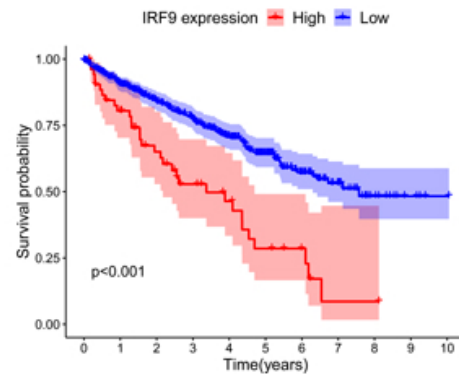

Supplement: Supplementary Figure 1 — The expression levels of the IRF family members between paired ccRCC samples and normal samples in the TCGA-KIRC dataset. [file DataSheet_1.zip › Supplementary figures/Supplementary Figure S3.pdf]

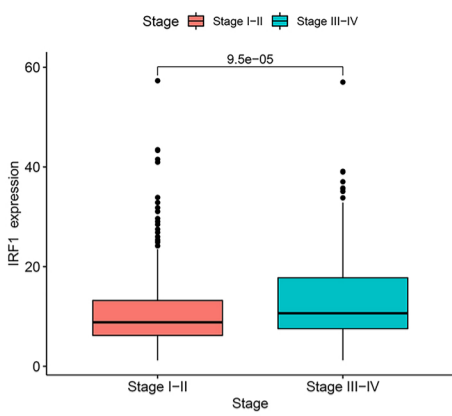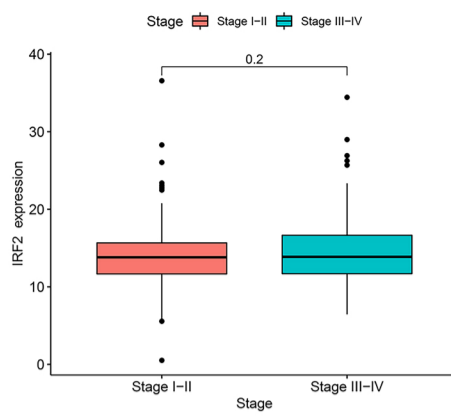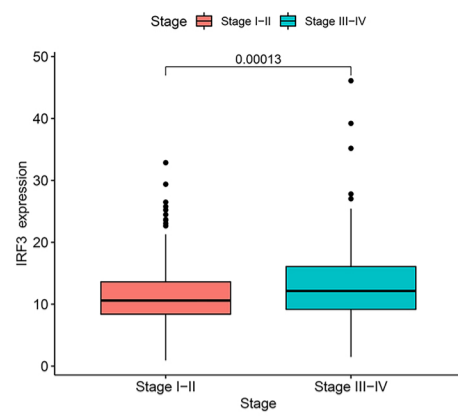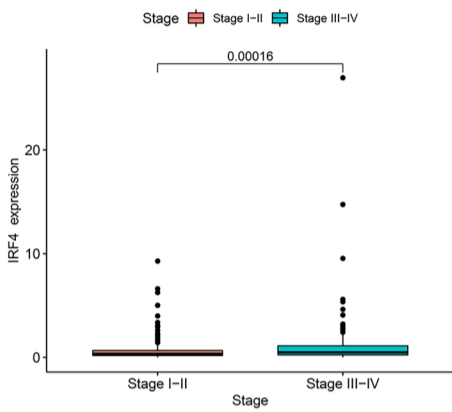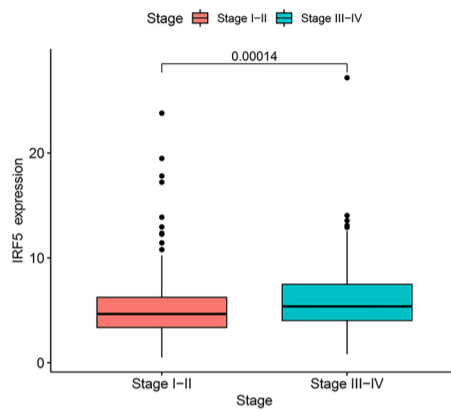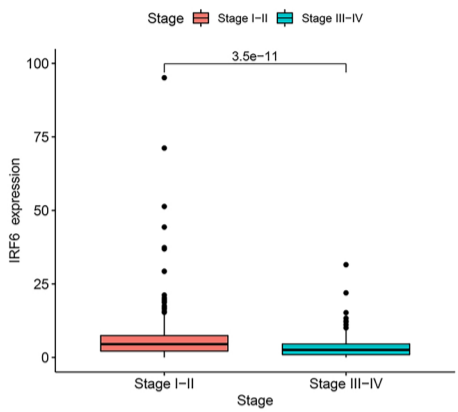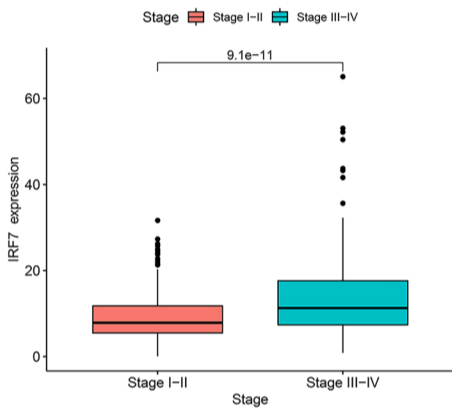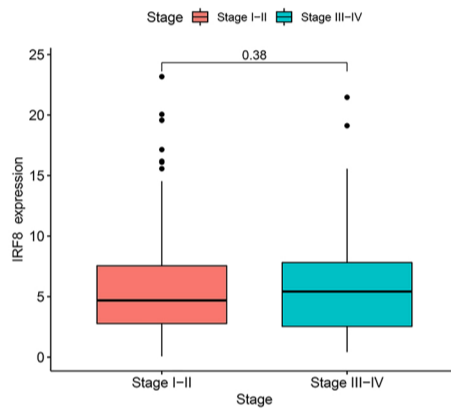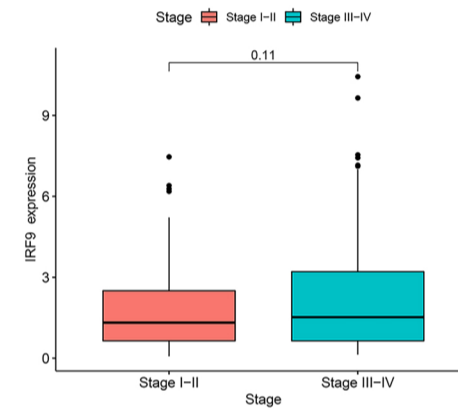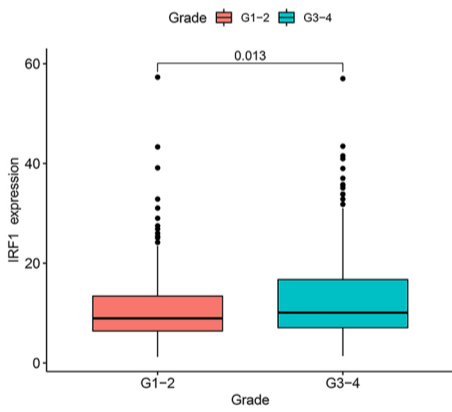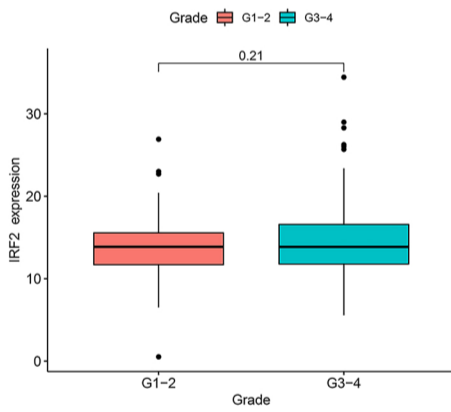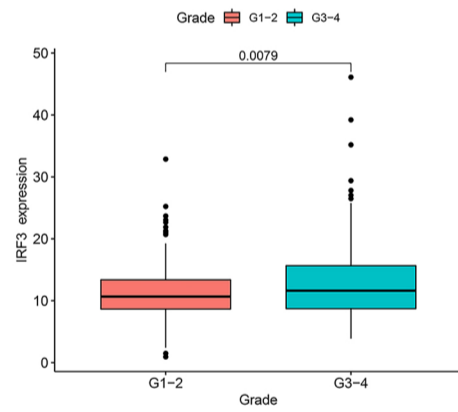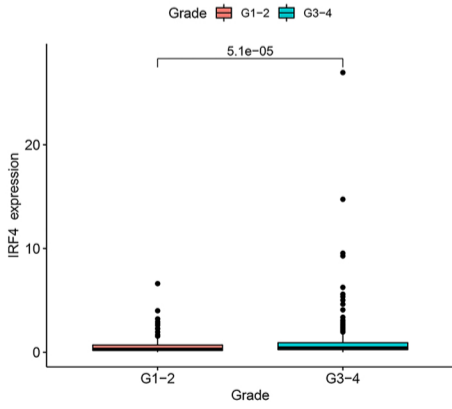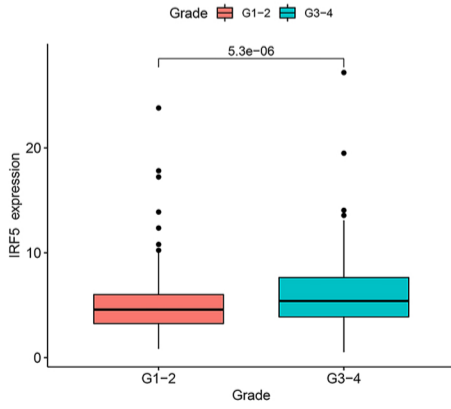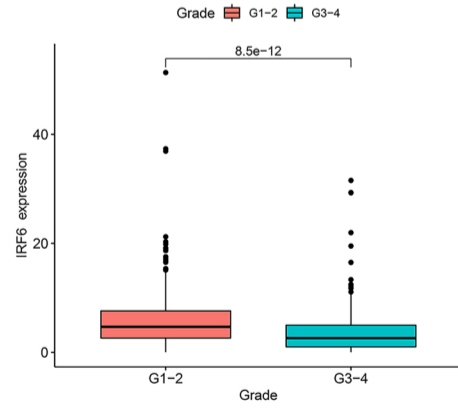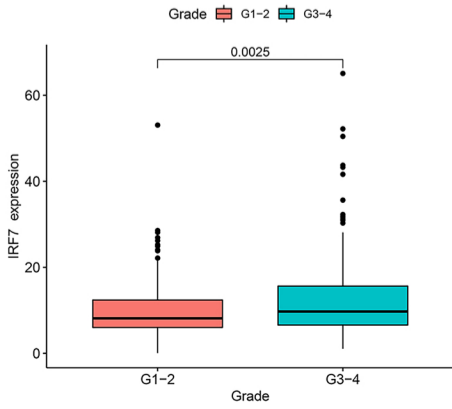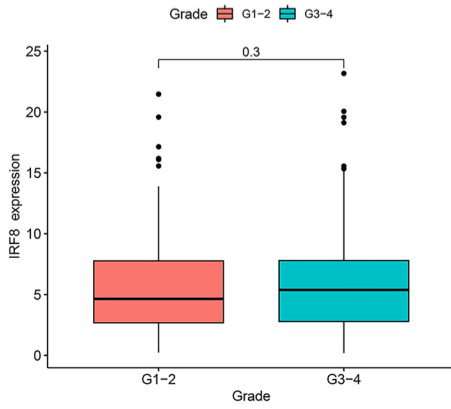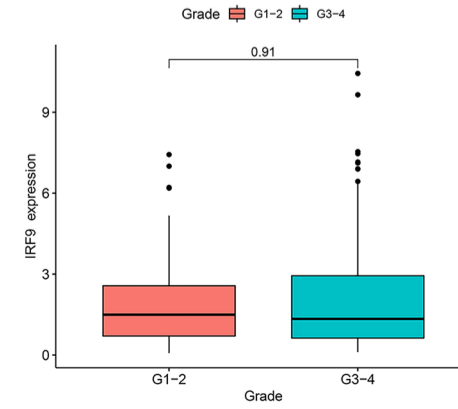

Supplement: Supplementary Figure 1 — The expression levels of the IRF family members between paired ccRCC samples and normal samples in the TCGA-KIRC dataset. [file DataSheet_1.zip › Supplementary figures/Supplementary Figure S4.pdf]

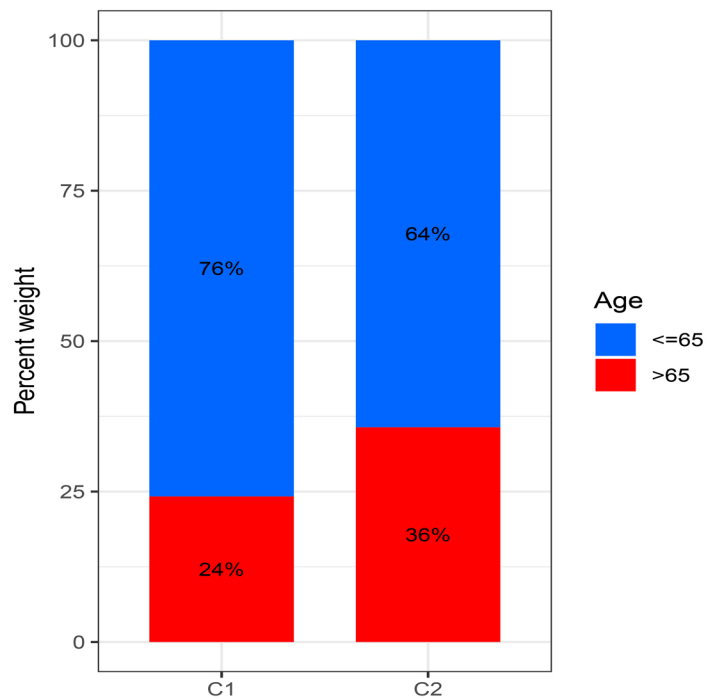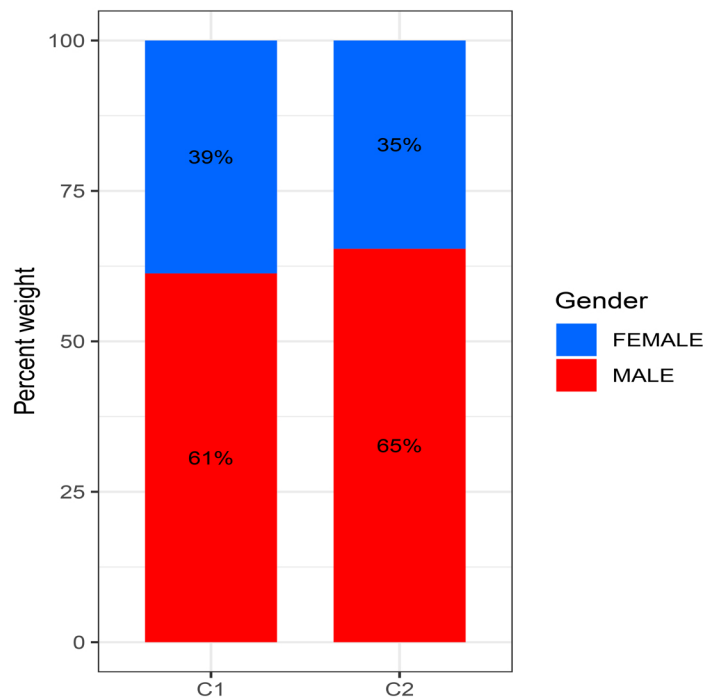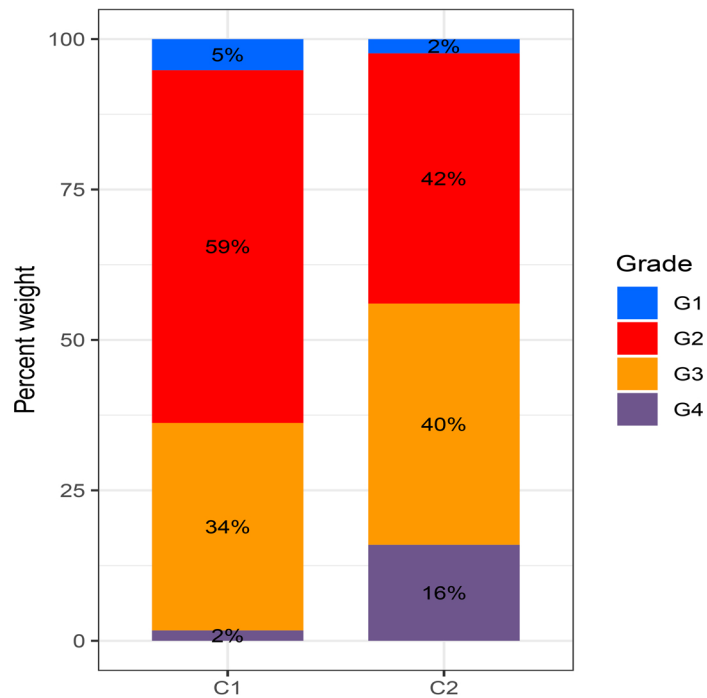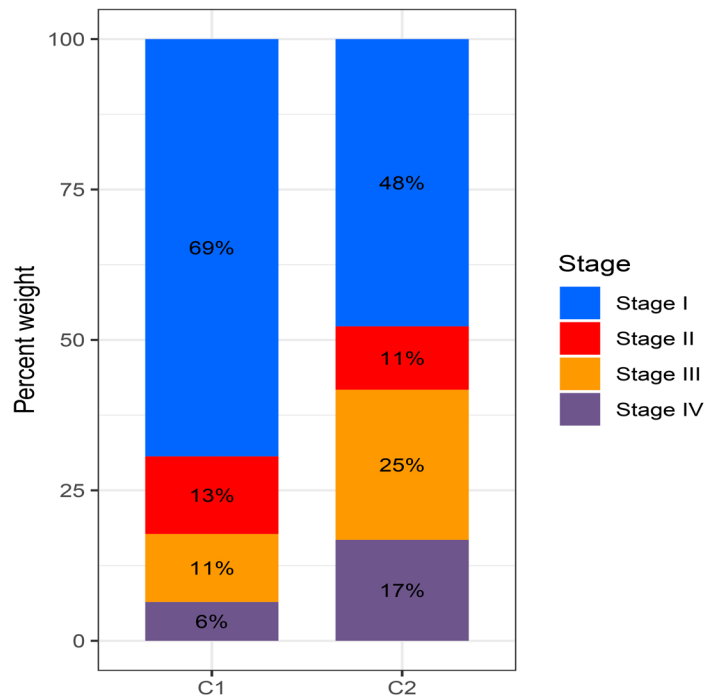

Supplement: Supplementary Figure 1 — The expression levels of the IRF family members between paired ccRCC samples and normal samples in the TCGA-KIRC dataset. [file DataSheet_1.zip › Supplementary figures/Supplementary Figure S6.pdf]

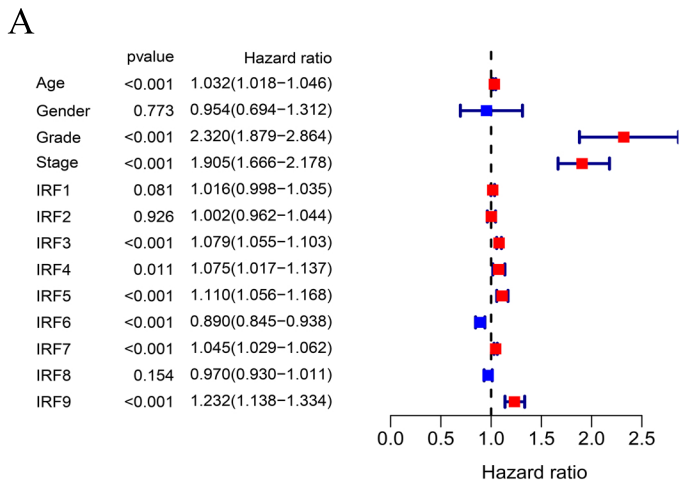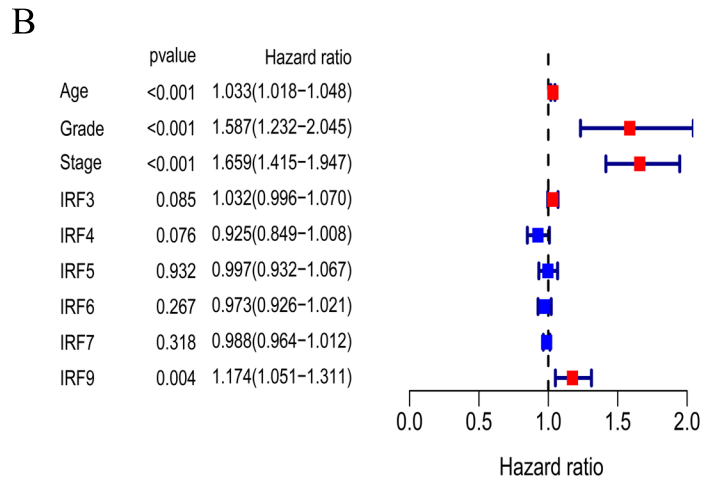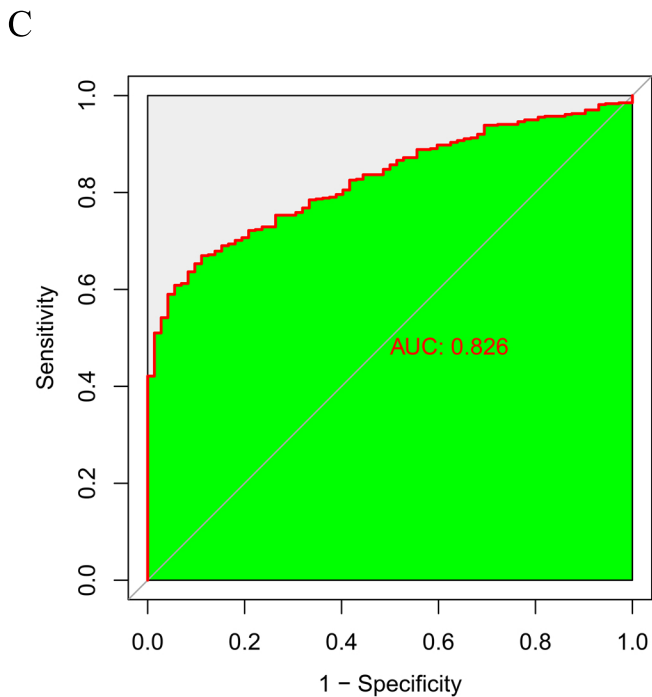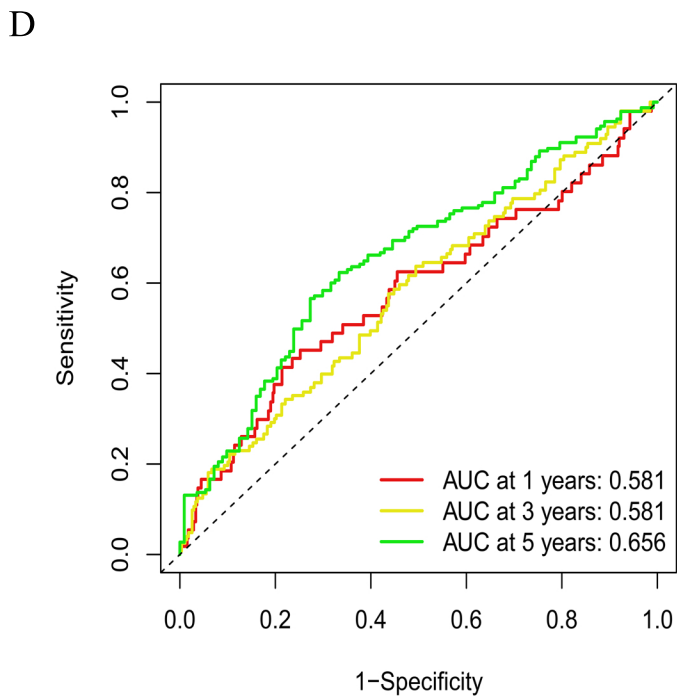

Supplement: Supplementary Figure 1 — The expression levels of the IRF family members between paired ccRCC samples and normal samples in the TCGA-KIRC dataset. [file DataSheet_1.zip › Supplementary figures/Supplementary Figure S5.pdf]

A

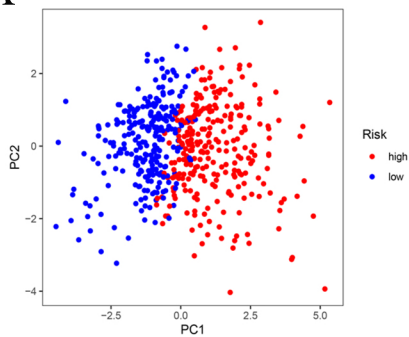

B

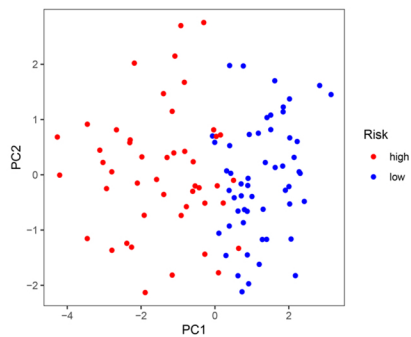

C

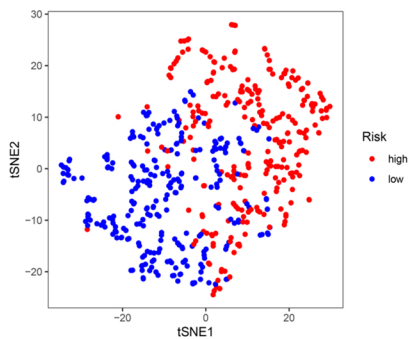

D

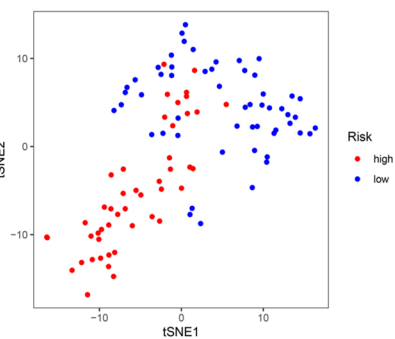

E

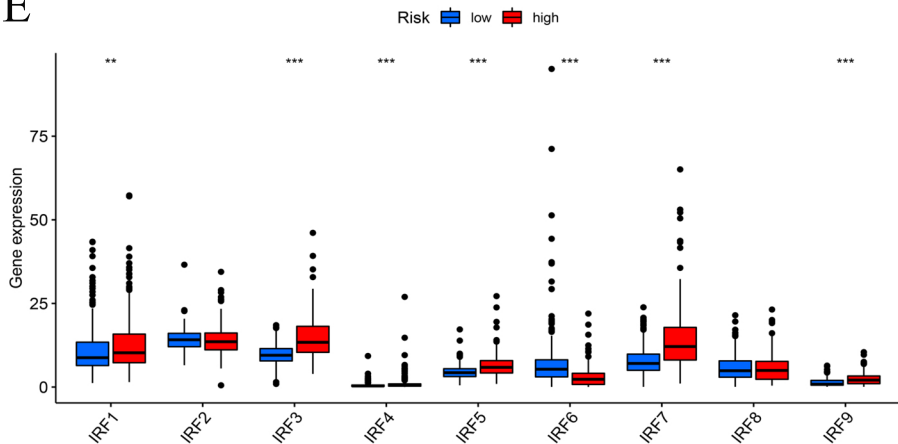

Supplement: Supplementary Figure 1 — The expression levels of the IRF family members between paired ccRCC samples and normal samples in the TCGA-KIRC dataset. [file DataSheet_1.zip › Supplementary figures/Supplementary Figure S7.pdf]

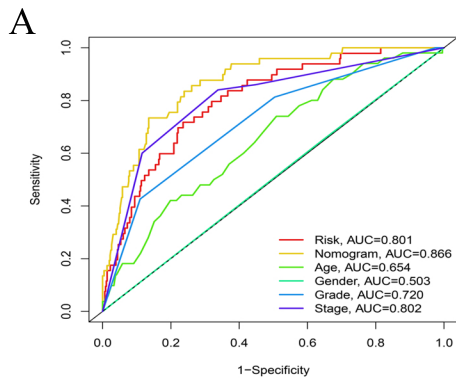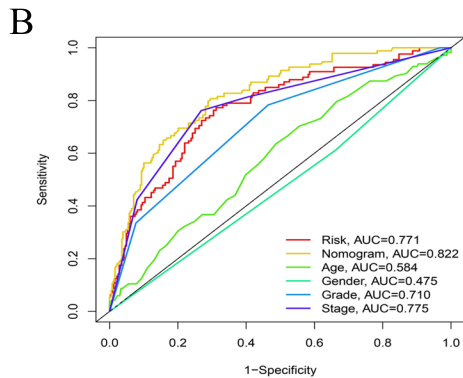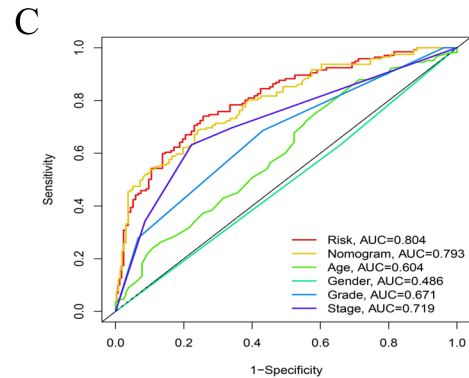

**D**

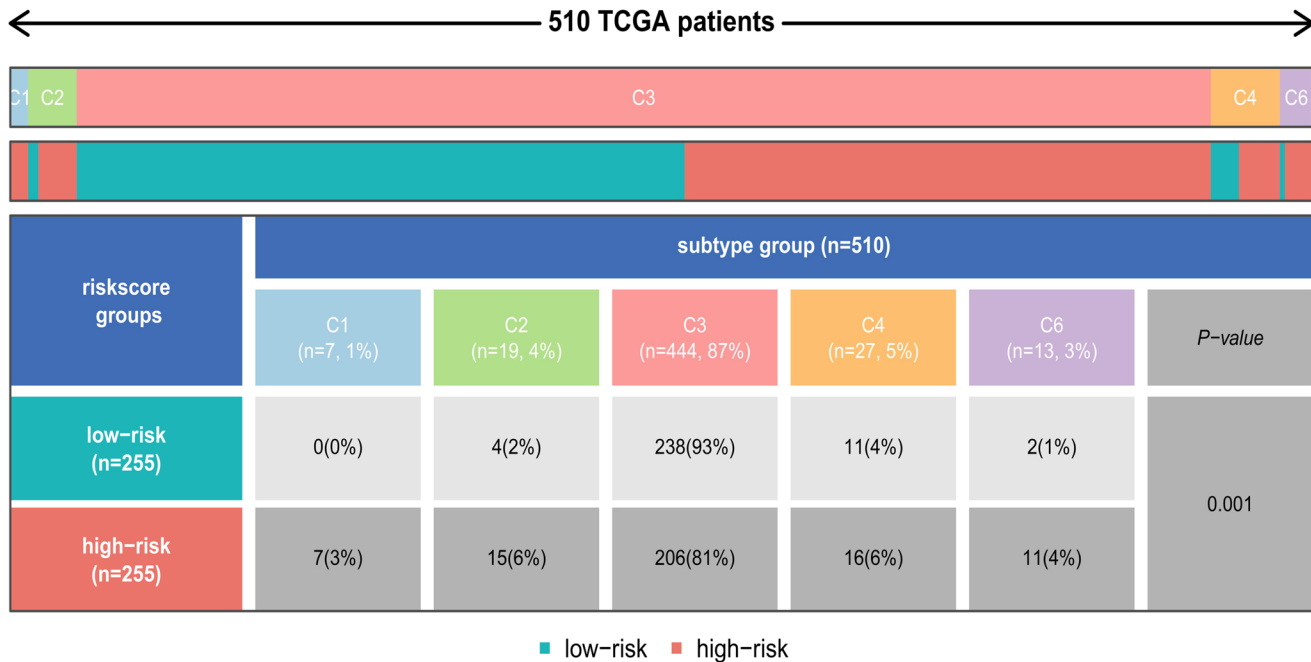

Supplement: Supplementary Figure 1 — The expression levels of the IRF family members between paired ccRCC samples and normal samples in the TCGA-KIRC dataset. [file DataSheet_1.zip › Supplementary figures/Supplementary Figure S8.pdf]

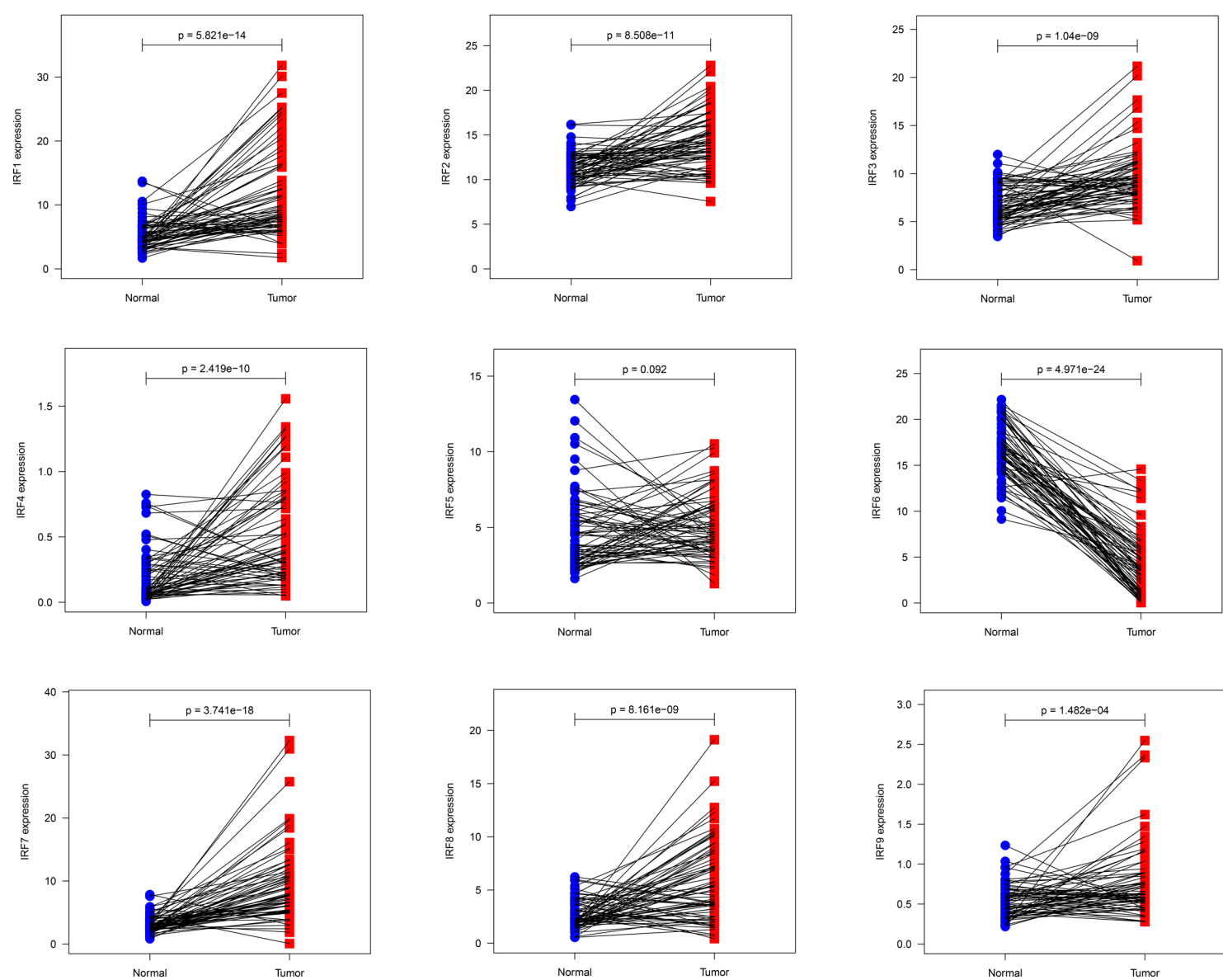

Supplement: Supplementary Figure 1 — The expression levels of the IRF family members between paired ccRCC samples and normal samples in the TCGA-KIRC dataset. [file DataSheet_1.zip › Supplementary figures/Supplementary Figure S1.pdf]
